# A Distributional Response Time Analysis of the Perceptual Disfluency Effect

**DOI:** 10.5334/joc.469

**Published:** 2025-10-16

**Authors:** Jason Geller, Pablo Gomez, Erin Buchanan, Dominique Makowski

**Affiliations:** 1 Department of Psychology and Neuroscience, Boston College, US; 2 Department of Psychology, Skidmore College, US; 3 Analytics, Harrisburg University of Science and Technology, US; 4 School of Psychology, University of Sussex, UK

**Keywords:** Disfluency, LDT, DDM, Ex-Gaussian, Distributional analyses, Word Recognition, Learning and memory

## Abstract

Perceptual disfluency, induced by blurring or difficult-to-read typefaces, can sometimes enhance memory retention, but the underlying mechanisms remain unclear. To investigate this effect, we manipulated blurring levels (clear, low-blur, high-blur) during encoding and assessed recognition performance in a surprise memory test. In Experiments 1A and 1B, response latencies from a lexical decision task were analyzed using ex-Gaussian distribution modeling and supplemented by drift diffusion modeling. Results showed that blurring differentially influenced parameters of the model, with high-blur affecting both early and late-stage processes, while low-blur primarily influenced early-stage processes. Recognition test results further revealed that high-blur words were remembered better than both clear and low-blurred words. Experiment 2 employed a semantic categorization task with a word frequency manipulation to further examine the locus of the perceptual disfluency effect. Similar to Experiments 1A and 1B, high-blur influenced both early and late-stage processes, while low-blur primarily affected early-stage processes. Low-frequency words exhibited greater shifting and skewing in distributional parameters, yet only high-frequency, highly blurred words demonstrated an enhanced memory effect. These findings suggest that both early and late cognitive processes contribute to the mnemonic benefits associated with perceptual disfluency. Overall, this study demonstrates that distributional and computational analyses provide powerful tools for dissecting encoding mechanisms and their effects on memory, offering valuable insights into models of perceptual disfluency.

We live in a world that is, even for adults, “blooming and buzzing with confusion” (James, 1890, p. 488). Yet we can still decipher cursive writing or follow conversations in noisy bars. This ability to cope with a noisy, confusing environment has long been studied at the intersection of education and cognitive psychology. Decades of work show that encoding difficulty can enhance long-term memory. Although people often assume that easier learning is better, many findings demonstrate the opposite: under certain conditions, making learning more effortful can improve retention. This phenomenon, known as the *desirable difficulties* principle (Bjork & Bjork, 2011), includes robust effects such as spacing study sessions (Carpenter et al., 2022), interleaving concepts rather than blocking them (Rohrer & Taylor, 2007), and generating or retrieving information instead of simply re-reading it (Roediger & Karpicke, 2006; Slamecka & Graf, 1978).

One straightforward example involves altering the perceptual characteristics of study materials to make them harder to process. A growing literature shows that such manipulations can improve memory (e.g., Geller et al., 2018; Geller & Peterson, 2021; Halamish, 2018; Rosner et al., 2015), a benefit referred to as the *perceptual disfluency effect* (see Geller et al., 2018).

## The Perceptual Disfluency Effect

The link between perceptual disfluency and memory dates back to the late 1980s. Nairne (1988), using the term perceptual-interference effect, employed backward masking with hash marks (e.g., ####) to make word encoding more difficult. Since then, a range of manipulations has been shown to elicit similar effects, including high-level blurring (Rosner et al., 2015), word inversion (Sungkhasettee et al., 2011), small text size (Halamish, 2018), handwritten cursive (Geller et al., 2018), and unusual typefaces (Geller & Peterson, 2021; Weissgerber & Reinhard, 2017; Weltman & Eakin, 2014).

Because these manipulations are simple to implement, researchers quickly began touting their educational potential. Interest grew following Diemand-Yauman et al. (2011), who reported that presenting material in disfluent typefaces (e.g., Comic Sans, Bodoni MT, Haettenschweiler, Monotype Corsiva) enhanced memory both in the lab and in high school classrooms across multiple content areas.

However, evidence for the effect has been inconsistent. A striking example is Sans Forgetica, a font designed to promote memory through slanted, gapped letters, forcing individuals to “generate” the missing parts of each word (Earp, 2018). Despite early claims, multiple studies have failed to replicate its benefits, finding it produces no memory benefit over and beyond normal fonts (Cushing & Bodner, 2022; Geller et al., 2020; Huff et al., 2022; Roberts et al., 2023; Taylor et al., 2020a; Wetzler et al., 2021). Similar null results have been reported for other perceptual manipulations like small fonts (Rhodes & Castel, 2008), degraded auditory stimuli (Rhodes & Castel, 2009), minor blurring (Yue et al., 2013), and alternative typefaces (Rummer et al., 2015).

Given these mixed findings, recent work has focused on discovering boundary conditions for the effect. Geller et al. (2018) showed a “Goldilocks” zone: memory benefits emerge only when stimuli are moderately, not excessively, difficult to read (e.g., easy-to-read cursive). Geller and Peterson (2021) further demonstrated that disfluency effects are stronger when test expectancy is low, reasoning that explicit test instructions lead participants to process all items deeply, reducing any added benefit of disfluency. Individual differences also play a role. For instance, Eskenazi and Nix (2021) found that strong spellers gained more from a disfluent font (i.e., Sans Forgetica) than weaker spellers.

Overall, perceptual disfluency can enhance memory in specific contexts but appears limited as an educational intervention, where students are typically aware of upcoming tests. Nonetheless, as Geller and Peterson (2021) argue, disfluency may hold practical value in everyday settings where memory is often incidental. The key challenge is to predict when and where such effects will reliably occur.

## Theoretical Accounts of the Disfluency Effect

To apply perceptual disfluency effectively in real-world settings, its underlying mechanisms must be better understood. Several theories have been proposed, with Geller et al. (2018) reviewing two major accounts. The metacognitive account (Alter et al., 2007; Pieger et al., 2016) views disfluency as a cue that prompts greater cognitive control and regulatory processing. Here, disfluency is detected after stimulus identification, and the specific type of disfluency is less important than the learner’s perception that material is difficult, which triggers regulatory processes. The compensatory processing account (Mulligan, 1996), on the other hand, is rooted in the interactive activation model of word recognition (McClelland & Rumelhart, 1981) and argues that disfluency enhances memory by recruiting top-down support from lexical and semantic representations. When input is noisy during encoding (e.g. by masking or blurring the stimulus), higher-level knowledge feeds back to aid recognition, and this deeper processing strengthens memory.

More recently, Ptok and colleagues proposed a limited-capacity, stage-specific model (Ptok et al., 2019; Ptok et al., 2020). They showed that memory benefits from encoding conflict depend on (1) the processing level engaged by the task and (2) metacognitive monitoring and control. Across six experiments, they found improved recognition when target words were paired with incongruent semantic distractors (e.g., *Chair – Alive* vs. *Chair – Inanimate*), but not with incongruent response distractors (e.g., *Lisa – left/right*). Both conditions slowed responses, but only semantic conflict boosted memory, suggesting the effect arises when tasks emphasize meaning. Pupillometry, a measure of cognitive effort (see Mathôt, 2018; van der Wel & Steenbergen, 2018 for reviews), confirmed that both types of conflict increase effort; yet, only semantic conflict translated into memory benefits. Importantly, the effect vanished when endogenous attention was manipulated (e.g., by using a chin-rest), mirroring perceptual disfluency findings. That is, disfluency benefits are eliminated when attention is manipulated by increasing test expectancy—for example, by telling participants they will be tested or by asking them to estimate how likely they are to remember an item on a later test (Besken & Mulligan, 2013; Geller & Peterson, 2021; Rosner et al., 2015).

Together, these accounts highlight different loci for the disfluency effect. The metacognitive account situates it post-lexically, after word recognition. The compensatory account links it directly to recognition, with disfluent words receiving more top-down support. The stage-specific model associates it with semantic-level processing, while also incorporating attentional and control processes that modulate when disfluency effects appear.

## Moving Beyond the Mean: Modeling Reaction Time (RT) Distributions

### Ex-Gaussian Distribution

To test the stages involved in the perceptual disfluency effect, researchers need methods that provide a finer-grained analysis of encoding. In learning and memory research, differences between fluent and disfluent conditions are typically assessed with mean reaction times (e.g., Geller et al., 2018; Geller & Peterson, 2021; Rosner et al., 2015). Although standard, mean RT analyses have been criticized for obscuring important distributional patterns (Balota & Yap, 2011).

RTs are typically unimodal, positively skewed, and often heteroscedastic, thereby violating assumptions of standard linear models (Wilcox, 1998). Reliance on mean RTs can obscure effects that selectively influence distributional shape—for example, the slow tail, which captures the longest responses, central tendency, or both. Moreover, because RTs reflect a mixture of decisional and non-decisional processes, mean RTs provide only limited leverage for isolating specific cognitive stages (Balota & Yap, 2011).

A widely used alternative is the ex-Gaussian distribution (Balota & Yap, 2011; Ratcliff, 1978), which decomposes RTs into three parameters: *μ* (mean of the Gaussian component, reflecting typical response speed), *σ* (its standard deviation), and *τ* (mean/SD of the exponential component, reflecting the slow tail). The overall mean equals *μ + τ*, and the SD is \[
\sqrt {{\sigma^2} + {\tau^2}}
\]. This decomposition allows researchers to distinguish between manipulations that shift the whole distribution, stretch the tail, or both.

For example, Heathcote et al. (1991) analyzed Stroop effects with an ex-Gaussian model. They found facilitation and interference effects on μ, interference on σ, and interference on *τ*. Mean RT analysis revealed only interference, as facilitation on μ and interference on τ canceled out—a finding hidden without distributional modeling.

Exploring effects from a distributional perspective has provided a richer understanding of how different experimental manipulations affect word recognition. Experimental manipulations can produce several distinct patterns. One pattern involves a shift of the entire RT distribution to the right, without increasing the tail or skew. A pattern such as this one would suggest a general effect and would manifest as an effect on *μ*, but not *τ*. As an example, semantic priming effects–where responses are faster to targets when preceded by a semantically related prime compared to an unrelated prime–can be nicely explained by a simple shift in the RT distribution (Balota et al., 2008). Alternatively, an experimental manipulation could produce a pattern where the RT distribution is skewed or stretched in the slower condition. This result suggests that the manipulation only impacts a subset of trials, and is discernable as an increase in *τ*.

An example of an effect that only impacts *τ* is the transposed letter effect in visual word recognition (Johnson et al., 2012). The transposed letter (TL) effect involves misidentification of orthographically similar stimuli that with transposed internal like, like mistaking “JUGDE” for “JUDGE” (Perea & Lupker, 2003). Finally, you could observe a pattern wherein an experimental manipulation results in both changes in *μ* and *τ*, which would shift and stretch the RT distribution. Recognizing low frequency words have been shown to not only shift the RT distribution, but also stretch the RT distribution (Andrews & Heathcote, 2001; Balota & Spieler, 1999; Staub, 2010).

Although largely descriptive, the model has been used to link parameters to processing stages. For instance, μ and σ have been tied to early, automatic processes such as spreading activation in semantic priming (Balota et al., 2008; Wit & Kinoshita, 2015). Conversely, τ has been linked to later, controlled processes involving attention and working memory (Balota & Spieler, 1999; Fitousi, 2020a; Kane & Engle, 2003). For example, τ differences in the transposed-letter effect have been attributed to post-lexical checking on a subset of trials (Johnson et al., 2012). Still, mapping distributional parameters onto cognitive processes remains debated and should be interpreted carefully (Heathcote et al., 1991; Matzke & Wagenmakers, 2009).

## Goals of the Present Experiments

In the present experiments, we pursued two aims related to perceptual disfluency. The first was to examine the replicability of the perceptual disfluency effect. To maximize the likelihood of observing this effect, we employed a manipulation that has previously been shown to enhance memory: perceptual blurring. Rosner et al. (2015) demonstrated across several studies that high-blur, but not low-blur can boost memory in a recognition memory test. Thus, not all disfluency manipulations are created equal (see Geller et al., 2018). Different perceptual manipulations affect processing in distinct ways, making it critical to identify which manipulations reliably produce disfluency effects and at what stage of processing. Following Rosner et al. (2015), we presented participants, with clear words (no blur), low-blurred words, and high-blurred words.

The second, more pivota, aim was to expand the methodological toolkit for investigating perceptual disfluency during encoding. To this end, we applied the ex-Gaussian distribution, which allows RT distributions to be decomposed into parameters reflecting different stages of processing. This approach offers a richer perspective beyond what mean response times alone can reveal. The ex-Gaussian model is widely used in the word recognition field (Balota et al., 2008), and its parameters are both interpretable and straightforward to implement. By using a distributional approach coupled with varying levels of perceptual disfluency, we aim to clarify the specific processing stages at which perceptual disfluency affects encoding, thereby providing a more mechanistic account of when disfluency enhances memory and when it does not.

### Predictions

Table 1 summarizes each theoretical account of perceptual disfluency and their predicted outcomes. Some of these accounts are articulated verbally and can be formalized in different ways. We made a good-faith effort to translate these verbal descriptions into models, while recognizing that reasonable researchers may make alternative modeling choices—an unavoidable reality of scientific inference (see McElreath, 2020). The ex-Gaussian distribution provides a descriptive framework for assessing how disfluency manipulations affect encoding. Each account makes specific predictions about the loci of the perceptual disfluency effect, which can be mapped onto model parameters:

1. If the metacognitive account (e.g., Alter et al., 2007; Pieger et al., 2016) holds, and the effect arises primarily at a post-lexical stage, one would expect a lengthening of the distribution tail (increases in *τ*) for blurred relative to clear words. Importantly, this perspective suggests that memory performance may not differ between high- and low-blurred words, given that perceptual disfluency is assumed to be largely subjective in nature.
2. In contrast, the compensatory processing account (Mulligan, 1996) would predict a shift in the distribution (increase in *μ*) for high-blurred words compared to low- and no-blurred words and better memory. Memory effects arising in this account are thought to be purely lexical/semantic. This expectation is in line with findings from Rosner et al. (2015), who reported that highly blurred words are associated with longer latencies, increased error rates, and better recognition memory.
3. If the disfluency effect reflects both early and late processing, the stage-specific account (Ptok et al., 2019; Ptok et al., 2020) predicts that high-blur (vs. clear/low-blur) will increase both *μ* (overall rightward shift) and *τ* (heavier tail). Similar encoding patterns have been observed with hard-to-read handwriting (e.g., Perea et al., 2016; Vergara-Martínez et al., 2021). Because low-blur is unlikely to recruit substantial post-lexical control, the account predicts no reliable change in either parameter for low-blur items.

**Table 1:** Mapping model predictions to theoretical constructs.


ACCOUNT	DESCRIPTION	LOCI	CONTRAST	EX-GAUSSIAN PREDICTIONS	QUANTILE PLOTS	RECOGNITION MEMORY PREDICTIONS

Meta-cognitive	Perceptual disfluency affects meta-cognitive processes via increased system 2 processing	Post-lexical	High-blur vs. Low-blur/Clear	μ: × *β/τ*: ↑	Late difference	High > Low/Clear

			Low-blur vs. Clear	μ: × *β/τ*: ↑	Late difference	Low > Clear

Compensatory-processing	Perceptual disfluency affects word recognition	Lexical/semantic	High-blur vs. Low-blur/Clear	μ: ↑ *β/τ*: ×	Complete shift	High > Low/Clear

			Low-blur vs. Clear	μ: × *β/τ*: ×	No difference	Low = Clear

Stage-specific	Disfluency effects rely on (1) the stage or level of processing tapped by the task and (2) monitoring and control processes	Lexical/semantic and Post-lexical	High-blur vs. Low-blur/Clear	μ: ↑ *β/τ*: ↑	Complete shift Shift + Late differences	High > Low/Clear

			Low-blur vs. Clear	μ: ↑ *β/τ*: ×	No difference	Low = Clear


*Note*. ↑ = higher estimate; ↓ = decrease estimate; x = no effect on parameter of interest.

## Experiment 1A: Context Reinstatement

In Experiment 1A, we collected RTs from a lexical decision task (LDT) during encoding followed by a surprise recognition memory test. Using a two-choice task, like the LDT, allowed us to examine how perceptual disfluency affects encoding processes using mathematical models. Based on previous work (Geller & Peterson, 2021), there was no mention of the recognition test when participants signed up for the study to give us the best chance of observing a disfluency effect.

### Method

#### Transparency and Openness

This study complies with transparency and openness guidelines. The preregistered analysis plan for this experiment can be found here: https://osf.io/q3fjn. All raw and summary data, materials, and *R* scripts for pre-processing, analysis, and plotting can be found at https://osf.io/6sy7k/.^1^ All deviations and changes from the preregistration are noted herein.

#### Participants

All participants were recruited through the Rutgers University subject pool (SONA system). We preregistered a sample size of 216 participants. A design of this size provides at least 90% power to detect effect sizes of *δ* ≥ 0.20, assuming a one-sided test with *α* = 0.05. A total of 263 participants completed the study. Per our exclusion criteria, 15 participants were removed for completing the experiment more than once, and 16 were removed for accuracy below 80%. No participants were excluded for being non-native English speakers or under 18 years of age. To account for oversampling and to ensure equal numbers across lists, we randomly selected 36 participants from each list, yielding a final sample of 216 participants. The study protocol was reviewed and approved by the Rutgers University Institutional Review Board.

#### Apparatus and stimuli

The experiment was run using PsychoPy software and hosted on Pavlovia (www.pavlovia.org). You can see an example of the experiment by navigating to this website: https://run.pavlovia.org/Jgeller112/ldt_dd_l1_jol_context.

We used 84 words and 84 nonwords for the LDT. Words were obtained from the *LexOPS* package (Taylor et al., 2020b). All of our words were matched on a number of different lexical dimensions. All words were nouns, 4–6 letters in length, had a known word recognition rate of 90–100%, had a low neighborhood density (OLD20 score between 1–2), high concreteness, imageability, and word frequency. Our nonwords were created using the English Lexicon Project (Balota et al., 2007). Stimuli can be found at our OSF project page cited above.

**Figure 1:**
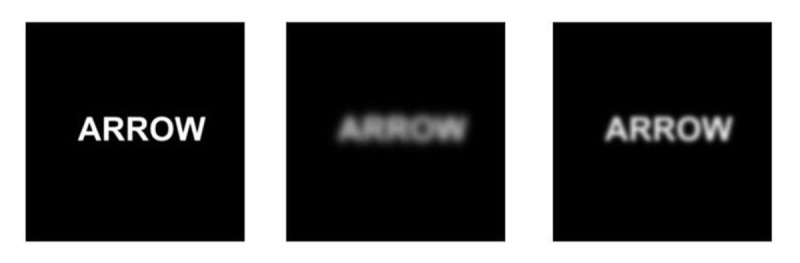
Clear (left), low-blur (10% blur) (right), and high-blur (15% blur) (center) examples.

**Blurring.** Blurred stimuli were processed through the {imager} package (Barthelme, 2023) and a personal script (https://osf.io/gr5qv). Each image was processed through a high-blur filter (Gaussian blur of 15) and low-blur filter (Gaussian blur of 10). These pictures were then imported into PsychoPy as picture files. See Figure 1 for examples how clear, low-blurred, and high-blurred words appeared in the experiment.

#### Design

We created two lists: 1) one list (84 words; 28 clear, 28 low-blur, and 28 high-blur) served as a study (old) list for the LDT task while the 2) other list served as a test (new) list (84 words; 28 clear, 28 low-blur, and 28 high-blur) for our recognition memory test that occurred after the LDT. We counterbalanced each list so each word served as an old word and a new word and were presented in clear, low-blurred, and high-blurred across participants. This counterbalancing resulted in six lists. Lists were assigned to participants so that across participants each word occurs equally often in the six possible conditions: clear old, low-blurold, high-blur old, clear new, low-blur new, and high-blur new. For the LDT task, we generated a set of 84 legal nonwords that we obtained from the English Lexicon Project. These 84 nonwords were used across all 6 lists.

#### Procedure

The experiment consisted of two phases: an encoding phase (LDT) and a test phase. During the encoding phase, a fixation cross appeared at the center of the screen for 500 ms. The fixation cross was immediately replaced by a letter string in the same location. To continue to the next trial, participants had to decide if the letter string presented on screen was a word or not by either pressing designated keys on the keyboard (“m” or “z”) or by tapping on designated areas on the screen (word vs. nonword) if they were using a cell phone/tablet. After the encoding phase, participants were given a surprise old/new recognition memory test. During the test phase, a word appeared in the center of the screen that either had been presented during study (“old”) or had not been presented during study (“new”). Old words occurred in their original typeface, and following the counterbalancing procedure, each of the new words was presented as clear, low-blurred, or high-blurred. All words were individually randomized for each participant during both the study and test phases and progress was self-paced. After the experiment, participants were debriefed. The entire experiment lasted approximately 15 minutes.

#### Data Analysis Plan

All models were fit in *R* (v. 4.5.1; R Core Team, 2025) using the Stan modeling language (Grant et al., 2017) via the {brms} package (Bürkner, 2017). We used maximal random-effects structures justified by the design (Barr et al., 2013).

We ran four chains of 5,000 MCMC iterations (1,000 warm-up), totaling 16,000 post-warm-up samples (except for the diffusion model, which used 2,000 iterations to reduce computation time). Model quality was checked via prior/posterior predictive checks, *R̂*, and effective sample size (ESS; Vehtari et al., 2021). Convergence was assessed using *R̂* (target ≤ 1.01) and effective sample size (ESS ≥ 1000) (Bürkner, 2017). Default (non-informative) priors were used for most parameters. Weakly informative priors were used for population-level parameters to enable Bayes factor (Evidence Ratio; ER) calculations for two sided-hypotheses against a point null. Full prior specifications are available in the Quarto source file on OSF: https://osf.io/6vew2.

We report posterior means and 90% credible intervals (CrIs) for one-sided hypotheses (preregistered differences), and 95% CrIs for two-sided hypotheses (against zero). Estimated marginal means were extracted using a combination of {emmeans} (Lenth, 2023) and {brms} (Bürkner, 2017). Additionally, we report the posterior probability that an effect lies in a particular direction and ER, which is a generalization of the Bayes factor for directional hypotheses.^2^ An ER > 3 indicates moderate to strong evidence for the hypothesis; ER < 0.3 indicates support for the alternative; and ER values between 0.3 and 3 are considered inconclusive. ERs were also used to assess point-null hypotheses (*δ* = 0). Hypotheses were considered supported if zero was excluded from the CrI, the posterior probability approached 1, and ER was > 3.

For all models, we applied ANOVA-style (effects) coding using contrast variables. For the Blur factor in Experiments 1A, 1B, and 2, we defined two orthogonal contrasts to capture the primary comparisons of interest. Contrast 1 compared high-blur against the average of clear and low-blur, coding high-blur as 0.5 and both clear and low-blur as –0.5. Contrast 2 isolated the difference between low-blur and clear, with low-blur coded as 0.5, clear as –0.5, and high-blur as 0. In Experiment 2, we also included a Frequency factor, with high-frequency coded as 0.5 and low-frequency as –0.5. Although these contrasts deviate from our preregistered comparisons, we believe they offer a more targeted test of our hypotheses. For transparency, we provide all pairwise comparisons in the accompanying visualizations

**Accuracy.** Accuracy (coded as correct [1] vs. incorrect [0]) was modeled using a Bayesian logistic regression with a Bernoulli distribution.

**Ex-Gaussian.** We modeled response times (in seconds) with an ex-Gaussian distribution,^3^ allowing the Gaussian mean/location (*μ*), the Gaussian standard deviation (*σ*) and the exponential scale (*β* = 1/*λ*) to vary by condition. Please note that when we refer to *β* we are referring to *τ* (*β/τ*). When fitting the ex-Gaussian distribution we use the identity link for *μ*, and the log link for *σ* and (*β/τ*).

**Quantile and Delta Plots.** In addition to ex-Gaussian analyses, we provide a graphical description of changes to the RT distribution using quantile and delta plots (Balota et al., 2008; De Jong et al., 1994). The process of visualization through quantile analysis can be broken down into four distinct steps:

1. Sorting and plotting: For correct trials, RTs are arranged in ascending order within each condition. We then plot the average of the specified quantiles (e.g., .1, .2, .3, .4, .5, .9).
2. Quantile averaging across participants: The individual quantiles for each participant are averaged, a concept reminiscent of Vincentiles.
3. Between-condition quantile averaging: The average for each quantile is computed between the conditions.
4. Difference calculation: We determine the difference between the conditions, ensuring the sign of the difference remains unchanged.

Typically, there are four observable patterns in the graphical depiction. No observable difference occurs when the conditions do not show any noticeable distinction. Late differences emerge when increasing differences appear later in the sequence, suggesting that the conditions diverge over time. A complete shift indicates a consistent difference across all quantiles, signaling an overall shift in the distribution. Finally, early differences reveal distinctions early in the reaction time distribution, suggesting an initial divergence between conditions.

**Recognition Memory.** Following recent trends (see Zloteanu & Vuorre, 2024), recognition memory was analyzed using a Bayesian generalized linear multilevel model (GLMM; a Bernoulli distribution with a probit link). Here the response of the participant (“say old” vs. “say new”) is modeled as a function of item status (“is old” vs. “is new”) and condition.

Bayesian GLMMs provide a more precise and flexible approach than traditional signal detection theory analyses. Following Signal Detection Theory (SDT; Green & Swets, 1966), participant responses can be classified as hits, correct rejections, misses, or false alarms, depending on the item status (“old” vs. “new”). In the probit regression framework, the interaction between item status and a predictor of interest corresponds directly to *d*′, while the main effects reflect response criterion (DeCarlo, 1998; for a detailed discussion of Bayesian SDT modeling see Zloteanu & Vuorre, 2024). Note that the model parameterization reflects *–c* (i.e., reversed sign) and this facet is what is reported in the paper. For visualization purposes, we use the conventional parameterization: positive values indicate more conservative responding, and negative values indicate a more liberal bias.

### Results

All models presented no divergences, and all chains mixed well and produced comparable estimates ( \[
{\hat R} < 1.01
\] and ESS > 1000).

#### Accuracy

The analysis of accuracy is based on 17,873 data points, after removing fast (< .2 s) and slow (> 2.5 s) RTs (2%). Model estimates can be found in Table 2. high-blur words had lower accuracy compared to clear and low-blurred words, *b* = –1.031, 90% CrI [–1.293, –0.77], ER = Inf. However, the evidence was weak for no significant differences in the identification accuracy between clear and low-blurred words, *b* = 0.041, 90% CrI[–0.216, 0.297], ER = 1.257.

**Table 2:** Posterior distribution estimates for accuracy model (Experiments 1A and 1B).


EXPERIMENT	HYPOTHESIS	MEAN	SE	CrI*	ER	POSTERIOR PROB

Experiment 1A	High-blur < (Low-blur + Clear)	–1.03	0.16	[–1.293, –0.77]	Inf	1.00

	Low-blur < Clear	0.04	0.13	[–0.216, 0.297]	1.26	0.56

Experiment 1B	High-blur < (Low-blur + Clear)	–1.10	0.17	[–1.376, –0.829]	Inf	1.00

	Low-blur = Clear	0.03	0.15	[–0.278, 0.322]	0.90	0.47


*Note*. CrI: 90% for one-sided tests and 95% for two-sided tests against 0. Posterior probability indicates the proportion of the posterior distribution that falls on one side of zero (either positive or negative), representing the probability that the effect is greater than or less than zero.

#### RTs: Ex-Gaussian

The analysis of RTs (correct trials and words) is based on 16,980 data points, after removing fast (< .2 s) and slow (> 2.5) RTs (1%).

A visualization of how blurring affected processing during word recognition can be seen in the quantile and delta plots in A summary of the ex-Gaussian model can be found in Table 3. Beginning with the μ parameter, there was greater shifting for high-blurred words compared to clear and low-blurred words, *b* = 0.107, 90% CrI [0.1, 0.114], ER = Inf. low-blurred compared to clear words showed greater shifting, *b* = 0.016, 90% CrI [0.012, 0.02], ER = Inf.

**Table 3:** Posterior distribution estimates for ex-Gaussian distribution (Experiments 1A and 1B).


EXPERIMENT	HYPOTHESIS	PARAMETER	MEAN	SE	CrI*	ER	POSTERIOR PROB

Experiment 1A	High-blur > (Low-blur + Clear)	Mu (*µ*)	0.11	0.00	[0.1, 0.114]	Inf	1.00

Experiment 1B	High-blur > (Low-blur + Clear)	Mu (*µ*)	0.12	0.01	[0.11, 0.127]	Inf	1.00

Experiment 1A	High-blur > (Low-blur + Clear)	Sigma (σ)	0.16	0.06	[0.057, 0.253]	163.95	0.99

Experiment 1B	High-blur > (Low-blur + Clear)	Sigma (σ)	0.32	0.07	[0.214, 0.43]	Inf	1.00

Experiment 1A	High-blur > (Low-blur + Clear)	Beta (*β/τ*)	0.43	0.04	[0.367, 0.487]	Inf	1.00

Experiment 1B	High-blur > (Low-blur + Clear)	Beta (*β/τ*)	0.38	0.03	[0.318, 0.43]	Inf	1.00

Experiment 1A	Low-blur = Clear	Sigma (σ)	0.03	0.05	[–0.066, 0.136]	16.22	0.94

Experiment 1B	Low-blur = Clear	Sigma (σ)	–0.09	0.06	[–0.212, 0.035]	5.92	0.85

Experiment 1A	Low-blur = Clear	Beta (*β/τ*)	–0.00	0.03	[–0.062, 0.061]	7.77	0.89

Experiment 1B	Low-blur = Clear	Beta (*β/τ*)	0.03	0.03	[–0.026, 0.084]	5.05	0.83

Experiment 1A	Low-blur > Clear	Mu (*µ*)	0.02	0.00	[0.012, 0.02]	Inf	1.00

Experiment 1B	Low-blur > Clear	Mu (*µ*)	0.01	0.00	[0.006, 0.015]	Inf	1.00


*Note*. CrI: 90% for one-sided tests and 95% for two-sided tests against 0. Posterior probability indicates the proportion of the posterior distribution that falls on one side of zero (either positive or negative), representing the probability that the effect is greater than or less than zero.

Analyses of the *σ* and *β/τ* parameters yielded a similar pattern. Variance was higher for high-blurred words compared to clear and low-blurred words, *b* = 0.157, 90% CrI [0.057, 0.253], ER = 163.948. Variance did not differ between low-blurred and clear words, *b* = 0.034, 90% CrI [–0.066, 0.136], ER = 16.22. There was greater skewing for high-blurred words compared to clear and low-blurred words, *b* = 0.427, 90% CrI [0.367, 0.487], ER = Inf. There was strong evidence for no difference between low-blurred and clear words, *b* = 0.00, 90% CrI [–0.062, 0.061], ER = 7.769.

#### Recognition Memory

**Figure 2:**
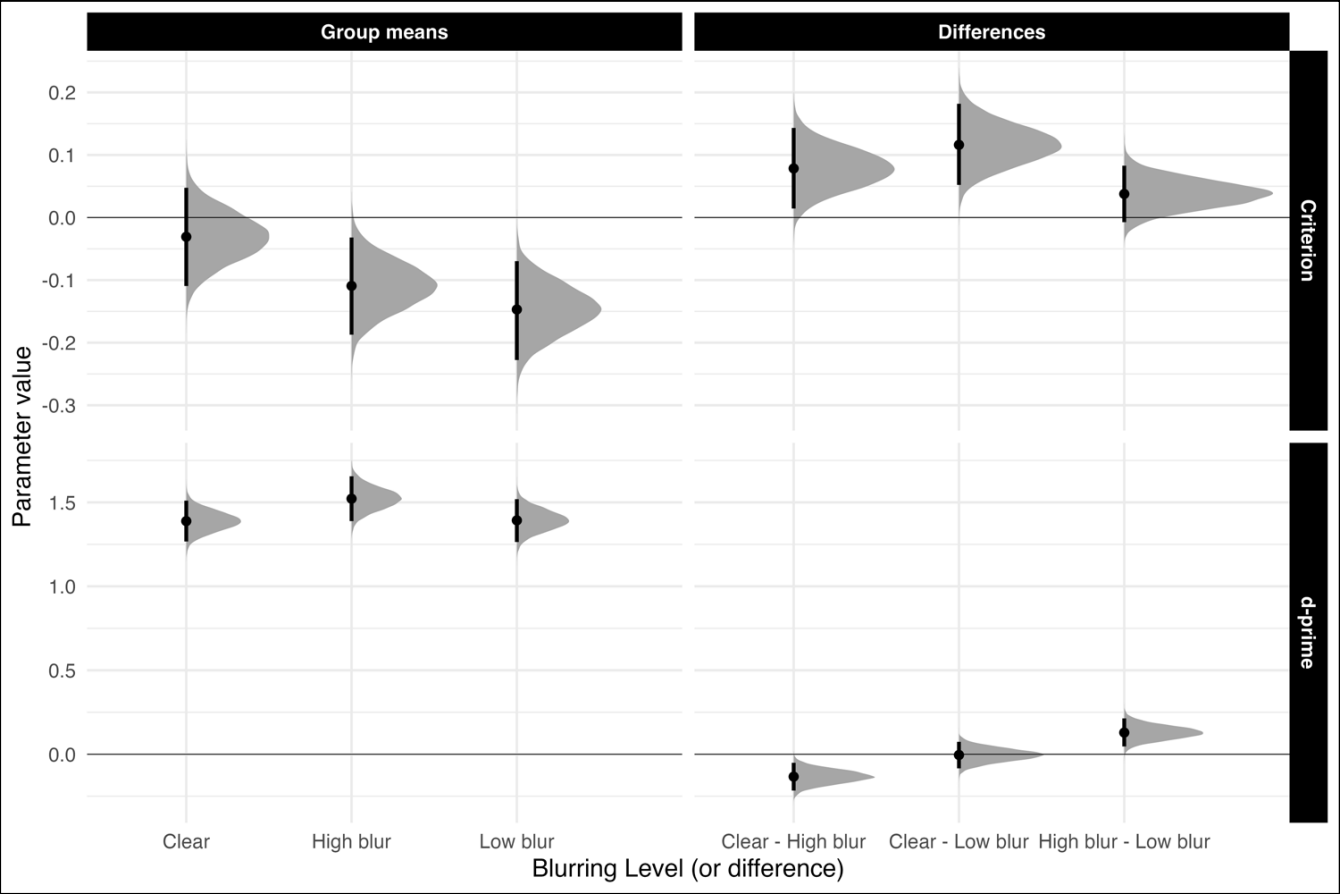
Estimated posterior distributions for d-prime and criterion, and differences, with 95% CrIs.

**Sensitivity.** Figure 2 highlights *d*′ and *c* means and comparisons across all groups. Sensitivity was higher for high-blurred words than for clear and low-blurred words, *β* = 0.131, 90% CrI [0.07, 0.193], ER = 7999. The evidence for no difference in sensitivity between clear words and low-blurred words was strong, *β* = 0.005, 90% CrI [–0.061, 0.072], ER = 1.194.

**Exploratory Analyses:** Bias. Low-blurred words had a bias towards more “old” responses compared to clear words, *β* = 0.116, 90% CrI [0.062, 0.171], ER = 2665.57. High-blurred words showed a slightly more liberal bias compared to clear and low-blurred words, *β* = 0.020, 90% CrI [–0.02, 0.06], ER = 4.22.

### Discussion

Experiment 1A successfully replicated the pattern of results found in Rosner et al. (2015). Specifically, we found high-blurred words had lower accuracy than clear and low-blurred words but had better memory.

### Distributional Modeling

**Figure 3:**
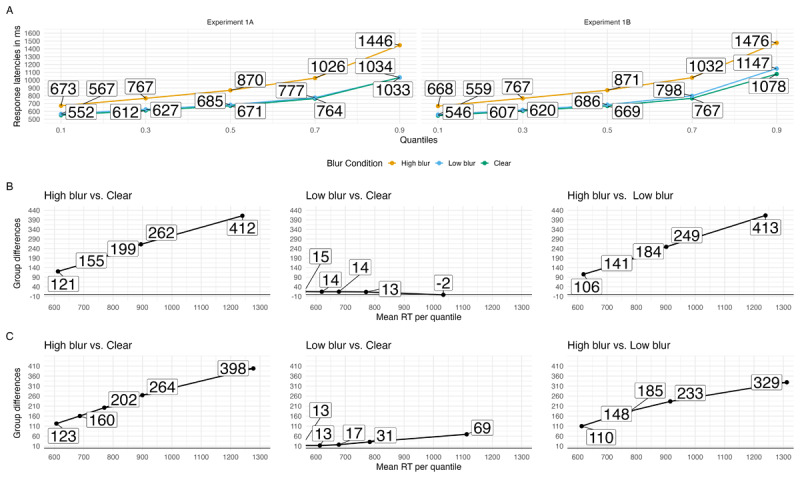
Quantile plots for each blur condition in Experiments 1A and 1B **(A)** and delta plots depicting the magnitude of the effect for hypotheses of interest over time in Experiments 1A **(B)** and 1B **(C)**. Each dot represents the mean RT at the .1, .3, .5, .7 and .9 quantiles.

Adding to these results, we used the ex-Gaussian distribution for modeling. Descriptively, high-blurred words induced a more pronounced shift in the RT distribution (*µ*) and exhibited a higher degree of skew (*β/τ*) compared to clear and low-blurred words. However, low-blurred words compared to clear words did not differ on *µ* or *β*. These patterns can be clearly seen in the quantile and delta plots in Figure 3.

This pattern argues against a purely metacognitive account (e.g., Pieger et al., 2016) and instead supports explanations that emphasize a combination of early and higher-level processing (e.g., stage-specific; Ptok et al., 2019), or compensatory processing (Mulligan, 1996)). However, considerable debate remains regarding the appropriateness of the ex-Gaussian distribution for drawing inferences about cognitive processes or stages (Fitousi, 2020a; Matzke and Wagenmakers, 2009).

#### DDM Results

Unlike the ex-Gaussian distribution, which makes little theoretical assumptions regarding process, the drift diffusion model (DDM) (see Ratcliff et al., 2016, for a comprehensive introduction) is a process-model, and its parameters can be linked to latent cognitive constructs (Gomez et al., 2013). The DDM is a popular computational model commonly used in binary speeded decision tasks such as the lexical decision task (LDT). The DDM assumes a decision is a cumulative process that begins at stimulus onset and ends once a noisy accumulation of evidence has reached a decision threshold. The DDM has led to important insights into cognition in a wide range of choice tasks, including perceptual-, memory-, and value-based decisions (Myers et al., 2022).

In the DDM, RTs are decomposed into several parameters that represent distinct cognitive processes. The most relevant to our purposes here are the drift rate (*v*) and non-decision time (ndt; *Ter*) parameters. Drift rate (*v*) represents the rate at which evidence is accumulated towards a decision boundary. In essence, it is a measure of how quickly information is processed to make a decision. A higher (more positive) *v* indicates a steeper slope, meaning that evidence is accumulated more quickly, leading to faster decisions. Conversely, a lower *v* indicates a shallower slope, meaning that evidence is accumulated more slowly. Drift rate is closely linked to the decision-making process itself and serves as an index of global processing demands imposed by factors such as task difficulty, memory load, or other concurrent cognitive demands—particularly when these processes compete for the same cognitive resources (Boag, Strickland, Loft, et al., 2019). Additionally, drift rates have been implicated as a key mechanism of reactive inhibitory control (Braver, 2012), where critical events (e.g., working memory updates or task switches) trigger inhibition of prepotent response drift rates (Boag, Strickland, Loft, et al., 2019; Boag, Strickland, Heathcote, et al., 2019). The *Ter* parameter represents the time taken for processes other than the decision-making itself. This includes early sensory processing (like visual or auditory processing of the stimulus) and late motor processes (like executing the response).

The DDM has been shown to be a valuable tool for studying the effects of different experimental manipulations on cognitive processes in visual word recognition. For example, Gomez and Perea (2014) demonstrated certain manipulations can differentially affect specific parameters of the model. For instance, manipulating the orientation of words (rotating them by 0, 90, or 180 degrees) affected the *Ter* component, but not *v* component. In contrast, word frequency (high-frequency words vs. low-frequency words) primarily influenced both the drift rate and non-decision time. These findings highlight the sensitivity of the DDM in identifying and differentiating the impact of various stimulus manipulations on different cognitive processes involved in decision-making.

We preregistered DDM analyses, and the model results can be found in the Appendix. Overall, we found high-blurred words impacted both an early non-decision component and a later, more analytic component evinced by higher *Ter* and a lower *v* than clear or low-blurred words. On the other hand, low-blurred words only affected *Ter*.

### Conclusion

Herein, we present evidence that different levels of disfluency can influence distinct stages of encoding, potentially contributing to the presence or absence of a mnemonic effect for perceptually blurred stimuli. Unlike most studies that commonly employ a single level of disfluency, our study incorporated two levels of disfluency. The results indicate that a subtle manipulation such as low-blur primarily affects early processing stages, whereas a more pronounced perceptual manipulation (i.e., high-blur) impacts both early and late processing stages. Regarding recognition memory, high-blurred stimuli were better recognized compared to low-blurred and clear words. This suggests that to observe a perceptual disfluency effect, the perceptual manipulation must be sufficiently disfluent to do so and tap later stages of encoding.

Given the important theoretical implications of these findings, Experiment 1B served as a conceptual replication. Due to the bias observed in the recognition memory test (i.e., low-blurred words were responded to more liberally), we do not present old and new items as blurred at test, instead all the words were presented in a clear, different, font at test.

## Experiment 1B: No Context Reinstatement

### Method

#### Transparency and Openness

This study was not preregistered. All raw and summary data, materials, and R scripts for pre-processing, analysis, and plotting for Experiment 1B can be found at https://osf.io/6sy7k/.

#### Participants

All participants were recruited through the Rutgers University subject pool (SONA system). We preregistered a sample size of 216 participants. A total of 282 participants completed the study. Per our exclusion criteria, 5 participants were removed for completing the experiment more than once, 19 were removed for accuracy below 80%, and 1 was removed for being under 18. No participants were excluded for being non-native English speakers. This resulted in 258 eligible participants. To account for oversampling and to ensure equal numbers across lists, we randomly selected 36 participants from each list, yielding a final analyzed sample of 216 participants. The study protocol was reviewed and approved by the Rutgers University Institutional Review Board.

#### Apparatus, Stimuli, Design, Procedure, and Analysis

Similar to Experiment 1A, the experiment was run using PsychoPy (Peirce et al., 2019) and hosted on Pavlovia (www.pavlovia.org). You can see an example of the experiment by navigating to this website: https://run.pavlovia.org/Jgeller112/ldt_dd_l1_jol.

We used the same stimuli from Experiment 1A. The main difference between Experiments 1A and 1B was all items were presented in a clear, Arial font. To make it more similar to Experiment 1A each set of words presented as clear, low-blur, and high-blur at study were yoked to a set of new words that were counterbalanced across lists. Therefore, instead of there being one false alarm rate there were 3, one for each blurring level. This ensured each word was compared to studied clear, studied high-blurred, and studied low-blurred words. The same model specifications and analyses used in Experiment 1A were used in Experiment 1B.

### Results

#### Accuracy

The analysis of accuracy is based on 17,809 data points, after removing fast (< .2 s) and slow (> 2.5 s) RTs (2%). A summary of posterior estimates are located in Table 2. high-blur words had lower accuracy compared to clear and low-blurred words, *b* = –1.101, 90% CrI [–1.376, –0.829], ER = Inf. However, the evidence was weak for no significant difference in the identification accuracy between clear and low-blurred words, *b* = 0.028, 90% CrI [–0.278, 0.322], ER = 0.9.

#### RTs: Ex-Gaussian

The analysis of RTs (correct trials and word stimuli) is based on 16,939 data points, after removing fast (< .2 s) and slow (> 2.5 s) RTs (2%). A visualization of how blurring affected processing during word recognition can be seen in the quantile and delta plots in Figure 3. A summary of the results can be found in Table 3. Beginning with the *µ* parameter, there was greater shifting for high-blurred words compared to clear and low-blurred words, *b* = 0.118, 90% CrI [0.11, 0.127], ER = Inf. low-blurred words had greater shifting compared to clear words, *b* = 0.01, 90% CrI [0.006, 0.015], ER = Inf. Analyses of the *σ* parameter yielded a similar pattern. Variance was higher for high-blurred words compared to clear and low-blurred words, *b* = 0.323, 90% CrI [0.214, 0.43], ER = Inf. There was no evidence of a difference in variance between low-blurred and clear words, *b* = –0.087, 90% CrI [–0.212, 0.035], ER = 5.918. Finally, looking at (*β/τ*) there was greater skewing for high-blurred words compared to clear and low-blurred words, *b* = 0.375, 90% CrI [0.318, 0.43], ER = Inf. There was no evidence of a difference in variance between low-blurred and clear words, *b* = 0.029, 90% CrI [–0.026, 0.084], ER = 5.052.

#### Recognition Memory

**Figure 4:**
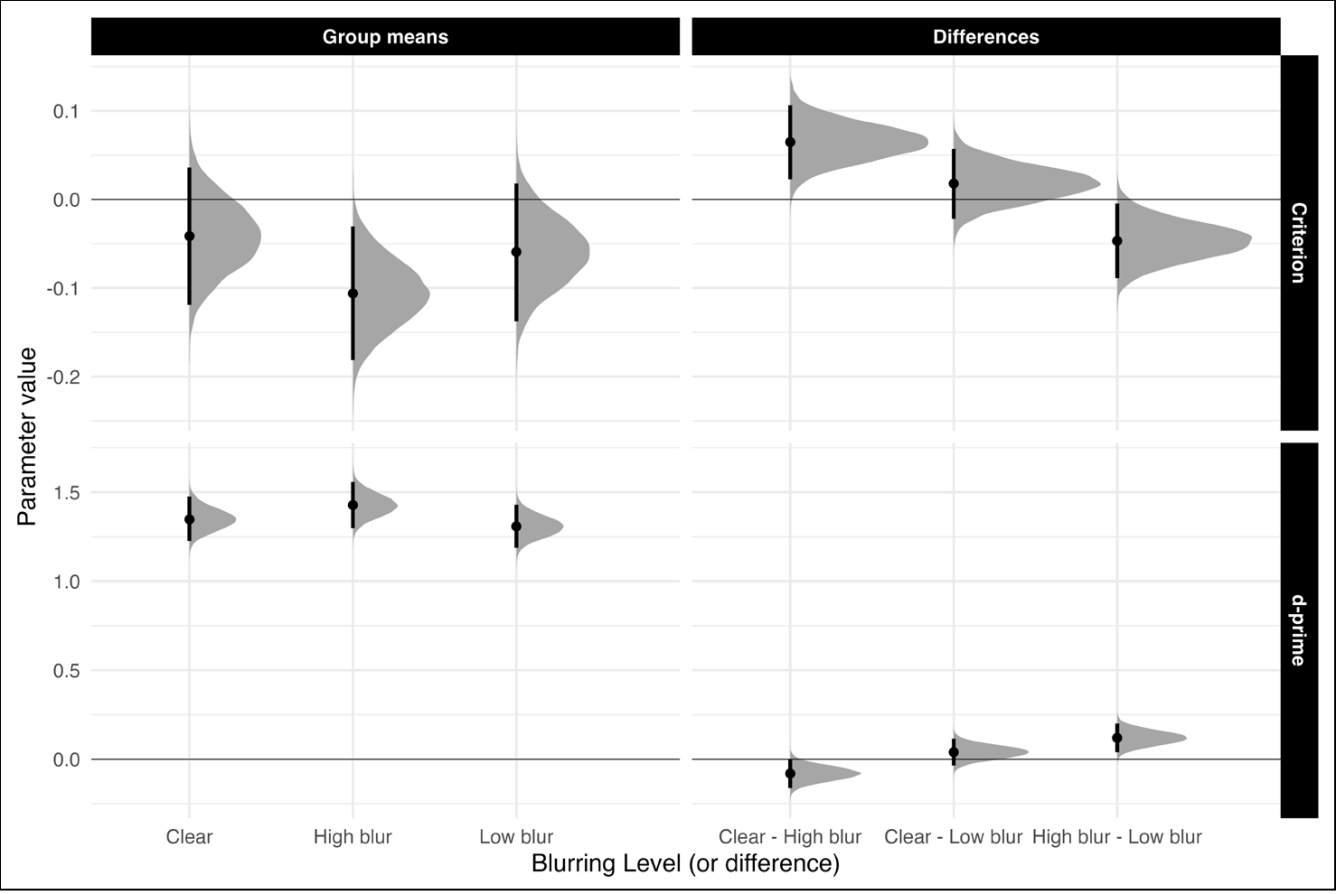
Estimated posterior distributions (mean) for d-prime and criterion, and differences, with 95% CrIs.

**Sensitivity.** Figure 4 highlights *d′* and *c* means and comparisons across all groups. Sensitivity was higher for high-blurred words than for clear and low-blurred words, *β* = 0.100, 90% CrI [0.040, 0.161], ER = 319. The evidence for no difference in sensitivity between clear words and low-blurred words was strong, *β* = –0.040, 90% CrI [–0.115, 0.036], ER = 1.455.

### Discussion

We replicated all findings from Experiment 1A in a design where blurring was not reinstated at test. In addition, DDM fits to both RTs and accuracy produced similar results (see Appendix).

## Experiment 2: Semantic Categorization

In Experiments 1A and 1B, we used the ex-Gaussian model to examine how visual blurring influences encoding and recognition memory. High-blurred words affected both early and late stages of processing, as indicated by an increase in shift and greater skew in the response time distribution. By contrast, low-blurred words, relative to clear words, appeared to influence only early-stage processing, primarily through distributional shifts.

Recognition memory paralleled this dissociation: sensitivity was greater for high-blurred words than for either clear or low-blurred words. These findings support a stage-specific account of the disfluency effect, suggesting that blurring influences not only early perceptual encoding but also later, higher-level processes involved in word recognition and memory.

To test this account more directly, we next examined whether blurring interacts with a higher-level linguistic variable that is known to affect both early and late stages of word recognition: word frequency. Numerous models of word recognition propose that lexical access varies systematically with frequency (e.g., Coltheart et al., 2001; McClelland & Rumelhart, 1981). Distributional analyses show that low-frequency words produce both larger shifts and greater skew than high-frequency words (Andrews & Heathcote, 2001; Balota & Spieler, 1999; Plourde & Besner, 1997; Staub, 2010; Yap & Balota, 2007; but see Gomez & Perea, 2014). If blurring indeed extends to higher-level stages of processing, then the combined effects of blurring and word frequency should provide a direct test of the stage-specific account.

In recognition memory, low-frequency words are generally remembered better than high-frequency words (Glanzer & Adams, 1985). This advantage has been attributed to the increased cognitive effort or attentional resources required to encode low-frequency items (Diana & Reder, 2006), a view referred to as the elevated attention hypothesis (Malmberg & Nelson, 2003; but see Pazzaglia et al., 2014 for alternative perspectives). Critically, in tasks such as semantic categorization and pronunciation, word frequency has been shown to interact with stimulus degradation, yielding over-additive effects (Yap & Balota, 2007). According to the logic of additive factors (Sternberg, 1969), such interactions suggest that the manipulated variables impact a shared processing stage. This interaction likely arises because perceptual disfluency disrupts early visual processing and lexical identification, thereby amplifying the frequency effect. Indeed, prior work has documented magnified word frequency effects under perceptual disfluency, including with handwritten cursive text (Barnhart & Goldinger, 2010; Perea et al., 2016) and rotated letter forms (Fernández-López et al., 2022).

In Experiment 2, we manipulated Word Frequency (high vs. low) and Blur (clear, low, high) within a semantic categorization task, followed by a surprise recognition test. The stage-specific account predicts that disfluency effects extend beyond perceptual encoding to influence later stages of processing. Thus, combining perceptual disfluency and lexical difficulty may particularly engage extra- or post-lexical mechanisms—especially for high-blurred, low-frequency words.

Individually, each factor may be resolved through more effortful lexical access (reflected in changes to *µ*). However, their combination could exceed lexical-level compensation, thereby recruiting additional control or decision-related processes. This account predicts an interaction on *β*, with blurred, low-frequency words producing especially long response-time tails, consistent with increased late-stage demands. Crucially, such late-stage engagement does not guarantee a memory advantage: when both perceptual and lexical demands are high, limited resources may be overtaxed, reducing or eliminating downstream mnemonic benefits.

With respect to memory, the stage-specific account further predicts that disfluency benefits are selective. Low-frequency words already attract increased attention during encoding (e.g., Kuchinke et al., 2007), making additional boosts from disfluency redundant. High-frequency words, by contrast, are typically processed more automatically and may gain more from disfluency manipulations that increase attention and depth of encoding. Supporting this view, prior work shows that disfluency effects on memory are strongest under otherwise fluent conditions (Ptok et al., 2019). When tasks already require sustained attention, the benefits tend to diminish—for example, when participants are stabilized with a chin rest (Ptok et al., 2020), warned of an upcoming memory test (Geller & Peterson, 2021), or required to spell rather than read words (Westerman & Greene, 1997).

Taken together, the stage-specific account would predict that blurring interacts with lexical difficulty to shape both response-time distributions and memory outcomes. Specifically, if blurring and word frequency jointly increase late-stage demands (indexed by *β*), downstream memory benefits should emerge only when sufficient cognitive resources remain—most likely for high-frequency words under otherwise fluent conditions. Experiment 2 tests these predictions by examining whether blur and frequency interact to influence early and late processing (ex-Gaussian parameters) and whether these effects translate into differences in subsequent memory.

### Method

#### Transparency and Openness

This study was preregistered https://osf.io/kjq3t. All raw and summary data, materials, and R scripts for pre-processing, analysis, and plotting for Experiment 2 can be found at our OSF page: https://osf.io/6sy7k/.

#### Participants

Participants were recruited via Prolific and compensated $12 per hour. Using Prolific’s built-in filters, we restricted eligibility to monolingual, native English–speaking Americans residing in the United States, with normal or corrected-to-normal vision. A total of 452 participants completed the study. Eleven were excluded for overall accuracy below 80%. One participant was excluded for being under 18 years of age. This left 440 participants. Consistent with Experiments 1A and 1B, we randomly sampled participants to reach our preregistered sample size of 432 and to have an equal number of participants per list. To be conservative and ensure adequate sensitivity to detect an attenuated interaction, we doubled the sample size of Experiments 1A and 1B and preregistered a target of 432 participants. The study protocol was reviewed and approved by the Princeton University Institutional Review Board.

#### Materials

One hundred and eighty words (half low-frequency and half high-frequency) were adapted from Fernández-López et al. (2022). We further selected an additional 45 animal words from their stimuli. To make the experiment more feasible for online participants and to balance our conditions, we split the remaining non-animal words and presented 90 (half high-frequency, half low-frequency) non-animal words along with the 45 animal words during the study phase. This split maintained the 2:1 ratio of non-animal to animal words used in previous experiments (e.g., Fernández-López et al., 2022; Perea et al., 2018).

At test, an additional 90 non-animal words not shown during the study phase served as “new” items in the recognition task. We created six counterbalanced lists to ensure that each word appeared as both “old” and “new,” and under each of the three blurring conditions (clear, high-blur, low-blur) across participants. Similar to nonwords in Experiments 1A and 1B, animal words were excluded from the final analysis. The animal word stimuli used by Fernández-López et al. (2022) varied in length (*M* = 5.3 letters; range: 3–9), but their average length closely matched that of the non-animal words (high-frequency: *M* = 5.3, range: 3–8; low-frequency: *M* = 5.3, range: 3–9). Animal words also covered a wide range of frequencies in the SUBTLEX database (*M* = 11.84 per million; range: 0.61–192.84).

#### Procedure

We used the same procedure as Experiments 1B. The main difference is that instead of making a word/non-word decision, participants made a semantic categorization judgement (i.e., “is an animal” or “is not an animal”). You can view the task here: https://run.pavlovia.org/Jgeller112/hf_lf_sem_1.

### Results

#### Accuracy

The accuracy analysis is based on 38,526 data points. After we removed fast (< .2 s) and slow (> 2.5 s) (1%). The full model summary for accuracy is presented in Table 4. We found strong evidence that high-blurred words were associated with lower accuracy compared to low-blurred and clear words, *b* = –0.914, 90% CrI [–1.263, –0.56], ER = Inf. The evidence for an accuracy difference between low-blurred and clear words was weak, *b* = –0.186, 90% CrI [–0.628, 0.203], ER = 0.571. The credible interval spanned zero, and the evidence ratio suggested only weak support for either hypothesis. The evidence for a frequency effect was similarly weak, *b* = 0.146, 95% CrI [–0.215, 0.548], ER = 0.665. Finally, interaction terms between blurring and word frequency yielded posterior distributions centered near zero, with 95% CrIs that included zero. Evidence ratios for these terms were close to 1, indicating substantial uncertainty and no clear preference for either the null or alternative hypothesis.

**Table 4:** Posterior distribution estimates for accuracy model (Experiment 2).


HYPOTHESIS	MEAN	SE	CrI*	ER	POSTERIOR PROB

High-blur < (Low-blur + Clear)	–0.91	0.21	[–1.263, –0.56]	Inf	1.00

Low-blur = Clear	–0.19	0.21	[–0.628, 0.203]	0.57	0.36

High frequency = Low frequency	0.15	0.20	[–0.215, 0.548]	0.67	0.40

Blur × Frequency (High vs. Low/Clear) = 0	–0.01	0.23	[–0.47, 0.438]	0.73	0.42

Blur × Frequency (Low vs. Clear) = 0	0.05	0.24	[–0.406, 0.554]	0.72	0.42


*Note*. CrI: 90% for one-sided tests and 95% for two-sided tests against 0. Posterior probability indicates the proportion of the posterior distribution that falls on one side of zero (either positive or negative), representing the probability that the effect is greater than or less than zero.

#### RTs: Ex-Gaussian

Given the complexity of the model, we employed stronger priors to facilitate convergence. For the *µ* parameter, we specified the same prior used in Experiments 1A and 1B. For the *β* parameter, we applied a more constrained prior: *Normal* (0,0.25). Default priors were retained for all remaining model parameters.

**Figure 5:**
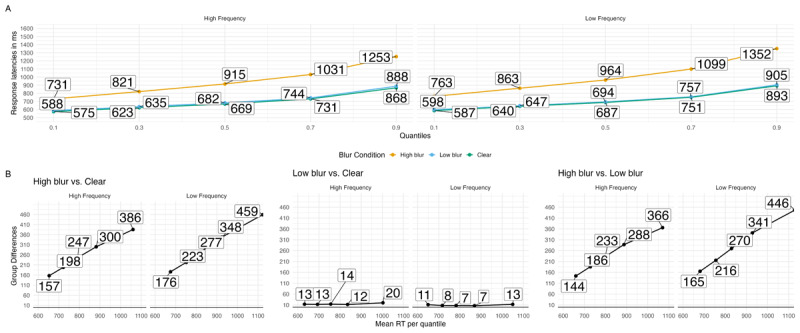
Group RT distributions in the blurring and word frequency manipulations in word stimuli. A. Quantile plots with each point represents the average RT quantiles (.1, .3, .5, .7, and .9) in each condition. B. Delta plots obtained by computing the quantiles for each participant and subsequently averaging the obtained values for each quantile over the participants and subtracting the values from each condition.

The analysis of RTs (correct trials and non-animal responses) is based on 37,823 data points, after removing fast (< .2 s) and slow (> 2.5 s) RTs (1%). Table 5 provides a model summary. Figure 5 visualizes RTs as quantile and delta plots, highlighting how blurring and word frequency affected processing during word recognition. High-blurred words showed larger central tendency shifts (*µ*) compared to the average of clear and low-blurred words, *b* = 0.172, 90% CrI [0.164, 0.18], ER = Inf. Low-blurred words also produced greater shifts relative to clear words, *b* = 0.010, 90% CrI [0.008, 0.013], ER = Inf.

**Table 5:** Posterior distribution estimates for ex-Gaussian distribution (Experiment 2).


HYPOTHESIS	PARAMETER	MEAN	SE	CrI*	ER	POSTERIOR PROB

high-blur > (vs. Clear/low-blur)	Mu (*µ*)	0.17	0.01	[0.164, 0.18]	Inf	1.00

low-blur > Clear	Mu (*µ*)	0.01	0.00	[0.008, 0.013]	Inf	1.00

High Frequency < Low frequency	Mu (*µ*)	–0.02	0.01	[–0.026, –0.011]	Inf	1.00

High-blur (vs. Low-blur/Clear) × Frequency	Mu (*µ*)	–0.01	0.01	[–0.02, 0.009]	1,024.59	1.00

Low-blur (vs. Clear) × Frequency	Mu (*µ*)	–0.00	0.00	[–0.009, 0.002]	1,453.96	1.00

High-blur > (vs. Clear/low-blur)	Sigma (σ)	0.64	0.04	[0.562, 0.706]	Inf	1.00

Low-blur < Clear	Sigma (*σ*)	–0.01	0.03	[–0.061, 0.045]	1.41	0.58

High frequency < Low frequency	Sigma (*σ*)	–0.05	0.04	[–0.108, 0.01]	11.05	0.92

High-blur (vs. low-blur/Clear) × Frequency	Sigma (*σ*)	0.08	0.07	[–0.031, 0.197]	7.67	0.89

Low-blur (vs. Clear) × Frequency	Sigma (*σ*)	–0.03	0.06	[–0.129, 0.065]	2.31	0.70

High-blur > (vs. Clear/low-blur)	Beta (*β/τ*)	0.55	0.03	[0.499, 0.603]	Inf	1.00

Low-blur > (vs. Clear)	Beta (*β/τ*)	–0.01	0.02	[–0.055, 0.027]	9.72	0.91

High frequency < Low frequency	Beta (*β/τ*)	–0.07	0.03	[–0.114, –0.016]	67.18	0.98

High-blur (vs. Low-blur/Clear) × Frequency	Beta (*β/τ*)	–0.14	0.05	[–0.222, –0.056]	332.33	1.00

Low-blur (vs. Clear) × Frequency	Beta (*β/τ*)	0.06	0.04	[0, 0.129]	19.52	0.95


*Note*. CrI: 90% for one-sided tests and 95% for two-sided tests against 0. Posterior probability indicates the proportion of the posterior distribution that falls on one side of zero (either positive or negative), representing the probability that the effect is greater than or less than zero. Sigma and Beta parameters are on the log scale.

There was a consistent word frequency effect: high-frequency words produced smaller shifts than low-frequency words, *b* = –0.018, 90% CrI [–0.026, –0.011], ER = Inf. Blur × Frequency interactions on *µ* were centered near zero, providing little evidence for an interaction. Specifically, the high-blur (vs. clear/low-blur) × Frequency interaction was *b* = –0.006, 90% CrI [–0.02, 0.009], ER = 1024.587, and the low-blur (vs. clear) × Frequency interaction was *b* = –0.004, 90% CrI [–0.009, 0.002], ER = 1453.958.

For variability (*σ*), responses to high-blurred words were more variable than those to clear and low-blurred words, *b* = 0.635, 90% CrI [0.562, 0.706], ER = Inf. The difference between low-blurred and clear words was weak, *b* = –0.007, 90% CrI [–0.061, 0.045], ER = 1.409. Frequency also modulated variability, with high-frequency words showing less variability than low-frequency words, *b* = –0.050, 90% CrI [–0.108, 0.01], ER = 11.048. Blur × Frequency interactions on *σ* provided mixed evidence: the high-blur (vs. clear/low-blur) × Frequency interaction was *b* = 0.083, 90% CrI [–0.031, 0.197], ER = 7.675, whereas the low-blur (vs. clear) × Frequency interaction was *b* = –0.031, 90% CrI [–0.129, 0.065], ER = 2.307.

Posterior estimates indicated greater skew (*β/τ*) for high-blurred words compared to clear and low-blurred words, *b* = 0.551, 90% CrI [0.499, 0.603], ER = Inf. The difference between low-blurred and clear words was negligible, *b* = –0.014, 90% CrI [–0.055, 0.027], ER = 9.724. Frequency robustly affected skew: high-frequency words showed less skew than low-frequency words, *b* = –0.065, 90% CrI [–0.114, –0.016], ER = 67.182. Evidence for interactions on skew was more nuanced. The high-blur (vs. clear/low-blur) × frequency interaction was supported, *b* = –0.139, 90% CrI [–0.222, –0.056], ER = 332.333. The low-blur (vs. clear) × frequency interaction suggested greater skewing for high-frequency words than for low-frequency words, although the CrI included 0, *b* = 0.064, 90% CrI [0, 0.129], ER = 19.525.

#### Recognition Memory

To aid in model convergence, we used a binomial (probit) family rather than a Bernoulli family when modeling our results as preregistered. While both approaches are appropriate for binary outcomes, the binomial model allowed us to aggregate responses within each condition, reducing the number of observations and easing computational demands. This aggregation improved sampling efficiency and stability, particularly given the complexity of the full factorial structure. However, as a trade-off, we were unable to model random intercepts and slopes for individual items, since aggregation collapses trial-level variability.

**Figure 6:**
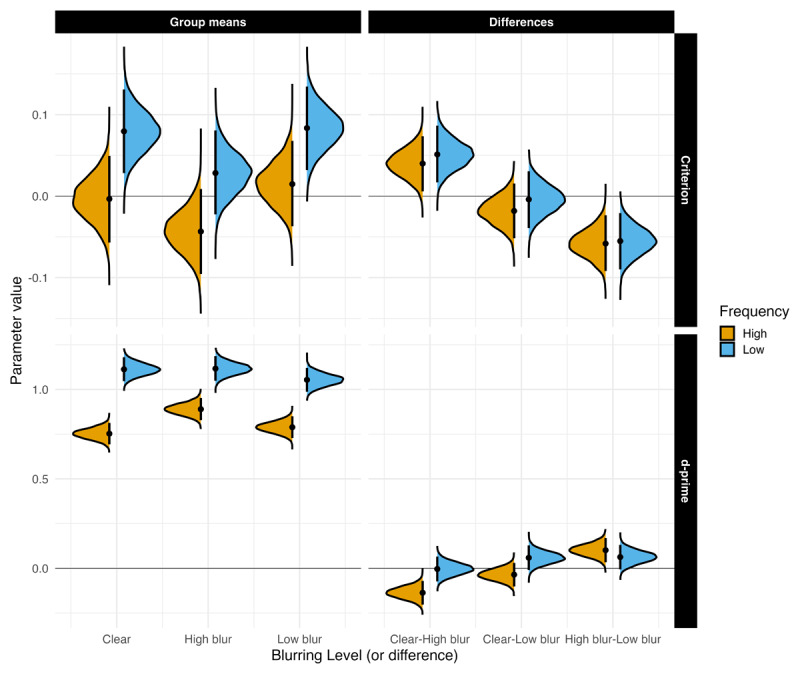
Estimated posterior distributions for d-prime and criterion, and differences between all conditions with 95% CrIs (thin lines).

**Sensitivity.** Consistent with Experiments 1A and 1B, sensitivity in recognition memory was higher for high-blurred words compared to both clear and low-blurred words (see Figure 6), *β* = 0.076, 90% CrI [0.041, 0.112], ER = 4999. The difference in recognition between clear and low-blurred words was negligible, with strong evidence in favor of the null hypothesis: *β* = –0.012, 95% CrI [–0.06, 0.036], ER = 4.911.

Sensitivity was higher for low-frequency words compared to high-frequency words, *β* = –0.270, 90% CrI [–0.306, –0.234], ER = Inf. Crucially, there was strong evidence for an interaction between Blur and Frequency, such that high-frequency words, but not low-frequency words, showed a selective memory benefit under high-blurring: *β* = 0.086, 95% CrI [0.017, 0.155], ER = 47.544. A second interaction emerged for the comparison between low-blur and clear words: *β* = 0.095, 90% CrI [0.016, 0.174], ER = 37.911. In this case, recognition was also better for high-frequency words when stimuli were presented with low-blur.

### Discussion

Experiment 2 examined how later stages of processing contribute to the memory boost sometimes produced by disfluent stimuli, and how this interacts with word frequency. We combined a word frequency manipulation with a semantic categorization task and looked at the full distribution of response times.

We found that the word frequency effect was especially strong for high-blurred words (compared to clear or low-blurred words). This effect grew larger at the slower end of the response time distribution, producing a steeper slope across quantiles (see Table 6 and Figure 5). In terms of modeling, this was reflected in changes to the *β* parameter of the ex-Gaussian distribution. Put simply, when words were blurred and varied in frequency, readers showed more slow responses—consistent with greater demands on late-stage processing. Similar effects have been observed with other kinds of difficult-to-read stimuli, such as handwritten cursive (Barnhart & Goldinger, 2010; Vergara-Martínez et al., 2021).

**Table 6:** Mean response time (in ms) for the word frequency effects across the .1, .3, .5, .7, and .9 quantiles of the RT distribution as a function of blurring. These values correspond to the quantile effects for Experiment 2.


BLUR	0.1	0.3	0.5	0.7	0.9

Clear	12.10	17.17	18.59	19.24	25.06

High-blur	31.46	41.84	48.68	67.18	98.36

Low-blur	9.93	12.14	11.99	13.62	17.73


Turning to memory, both high- and low-blurred words produced a perceptual disfluency effect relative to clear words. However, the benefit depended on frequency. High-frequency words showed a memory advantage under both blur conditions. Low-frequency words, in contrast, did not benefit from blurring. This asymmetry is informative: in the RT data, high-blur produced a significant interaction on the *β* parameter, with low-frequency words showing a disproportionately long tail (slower responses) — a marker of greater late-stage processing difficulty. Yet this extra effort did not translate into better memory. By comparison, high-frequency words showed only modest increases in the tail but did reap a memory benefit.

Taken together, these findings suggest that greater late-stage processing (as reflected in longer response time tails) may be necessary but not sufficient for memory benefits from disfluency. In our study, high-blur exaggerated the word frequency effect on the tail of the distribution, indicating more prolonged or effortful processing. However, only under some conditions (e.g., high-frequency words) did this additional processing translate into improved memory performance.

Interestingly, low-blur also interacted with word frequency. High-frequency words showed a memory advantage under low-blur, even though the statistical model suggested only modest changes in the tail of the response time distribution. The evidence ratio indicated support for a positive effect, although the credible interval included zero. What matters for interpretation is the *direction*: unlike the high-blur condition, under low-blur it was the high-frequency words that showed more signs of late-stage processing than the low-frequency words.

Why might this be? For high-frequency words, low-blur may have disrupted the usually automatic recognition process just enough to slow people down and force some extra lexical or semantic processing. Rather than being harmful, this mild disruption could have deepened encoding, resulting in a memory benefit. By contrast, low-frequency words may not have been accessible enough to benefit from the same subtle increase in processing effort.

This pattern highlights that memory benefits from disfluency are not simply a matter of “more effort is better.” Instead, they appear when perceptual difficulty interacts with lexical accessibility in the right way. Modest disfluency can encourage extra elaboration of familiar words without overwhelming cognitive resources, leading to stronger memory. But when disfluency is too great, or when the items are already hard to process, the added effort may not yield any benefit.

## General Discussion

Interfering with stimulus perception during encoding can sometimes improve later explicit memory. The mixed data on perceptual disfluency has called into question the utility of such manipulations in the learning domain. One of the main aims of the current set of experiments was to examine the underlying mechanisms of the perceptual disfluency effect to better understand when perceptual disfluency aids memory and when it does not. To this end, our study delved into the impact of one type of perceptual disfluency–blurring (i.e., low-blurring and high-blurring)–on the process of encoding, as assessed through a LDT (Experiments 1A and 1B), and a semantic categorization task (Experiment 2). RT distributions were analyzed with an ex-Gaussian model (Experiments 1A, 1B, 2) and DDM (Experiments 1A and 1B). Application of this model offered a comprehensive descriptive and theoretical framework through which to examine the perceptual disfluency effect.

To recapitulate our findings, during encoding, high-blurred words showed greater distributional shifting and skewing compared to clear and low-blurred words. Conversely, low-blurred words compared to clear words showed greater distributional shifting, but there was no difference in skewing. Turning to recognition memory, high-blurred words were more likely to be recognized at test compared to clear words and low-blurred words. This pattern arose regardless if context was reinstated at test (Experiment 1B). This pattern replicates the results from Rosner et al. (2015). In addition, we showed word frequency (Experiment 2) also modulates the disfluency effect. Namely, high-blurred low-frequency words did not show a disfluency effect.

These findings have several implications. At a theoretical level, the current data suggests that for perceptual disfluency to benefit memory it must be disfluent enough to affect both early and late stages of processing. A manipulation that only produces a general slowing of responses is not sufficient to enact a mnemonic effect. However, an important caveat to this result is that processes during encoding of the word itself are not enough to produce a mnemonic benefit. In Experiment 2, we did not observe better memory for low frequency-high-blurred words which are the hardest and presumably receive the most top-down processing. We only observed a disfluency effect for high-blurred high-frequency words. This points to the importance of control processes and processing limitations in producing the disfluency effect.

We argue that the current findings align most closely with the stage-specific account proposed by Ptok et al. (2019). Although this account was originally developed to explain memory effects requiring conflict during encoding (e.g., semantic interference), it also provides a useful framework for understanding our results. Indeed, Ptok et al. (2019) and Ptok et al. (2020) suggested links between their framework, perceptual disfluency effects, and desirable difficulties more broadly. According to the stage-specific account, memory performance depends on the nature of processing during encoding and the deployment of cognitive control mechanisms.

In our experiments, participants judged whether letter strings were words or nonwords (Experiments 1A and 1B) or whether a word belonged to the animal category (Experiment 2). For skilled readers, such tasks are executed automatically and fluently. When paired with perceptual disfluency, this automaticity can lead to memory advantages for disfluent stimuli. However, when we manipulated word frequency, recognizing low-frequency words required greater effort and attentional resources on top of the perceptual disfluency introduced by blurring, further increasing task demands. These heightened processing requirements may have offset the potential benefits of blurring, as more resources were diverted to lexical access.

Evidence for this capacity-limited view comes from several sources. For example, Geller et al. (2018) showed that both easy-to-read and hard-to-read cursive words were remembered better than computer-print words, though the advantage was substantially larger for easier to read cursive words. Similarly, participants with lower working memory capacity benefit less from perceptual disfluency than those with higher capacity (Lehmann et al., 2015). At a broader level, Wenzel and Reinhard (2019) suggested that intelligence may moderate when desirable difficulties enhance learning.

Complementing this stage-specific perspective, Gagl et al. (2020) proposed a more stage-agnostic framework: the orthographic prediction error (OPE) model. Drawing on fMRI and EEG data, this model holds that reading involves generating visual–orthographic predictions and comparing them to incoming input. Visual degradation increases OPE—the mismatch between expected and observed letter input—and when predictions are impaired or suspended due to unpredictability, the benefits of top-down facilitation are reduced. Processing then relies more heavily on bottom-up input, which can slow responses and alter encoding quality. This framework helps clarify how perceptual disfluency influences both response-time distributions and memory outcomes, while situating the stage-specific account within a broader predictive-processing view. Future research may further elucidate how these perspectives converge.

At a methodological level, our experiments demonstrate that a straightforward blurring manipulation can benefit memory, which we observed whether or not we reinstated the context during testing. However, blurring has to be sufficiently difficult to do so. If the secondary task requires too much attentional control the effect might not be observed or attenuated.

More significantly, our current experiments underscore the benefits of using mathematical and computational models to examine stages or levels of processing during encoding. A frequent critique of the ex-Gaussian model is that it lacks a clear correspondence to specific cognitive constructs (Fitousi, 2020b). To address this limitation, we also fit a DDM to the encoding data from Experiments 1A and 1B (see Appendix A). Both models converged on similar findings: response time distributions were differentially affected by the degree of visual blur. Words with low-blur primarily influenced early or non-decision stages of processing whereas highly blurred words impacted both early and later stages. These findings suggest that both the ex-Gaussian and DDM are sensitive to perceptual disfluency and can help uncover underlying cognitive mechanisms during encoding (but see Hu et al., 2022 for a DDM account of perceptual disfluency at retrieval). Although the models converged on similar patterns, it remains an open question whether one should be favored over the other.

Furthermore, our distribution modeling of RTs appears to be a more sensitive method. Although we found weak evidence for differences between clear and low-blurred conditions in measures like accuracy, we did notice variations in non-decision time and a shift in the response time distribution for low-blurred words compared to clear words. We recommend that future studies employ distribution modeling and DDM to decompose response times and directly quantify the impact of perceptual disfluency on encoding.

Finally, at a practical level, our findings suggest that blurring can benefit later memory. However, several caveats should be noted. First, these experiments were conducted online using relatively simple materials (i.e., list learning). It remains unclear how well these effects would generalize to classroom settings or more educationally realistic materials and designs (see Geller & Peterson, 2021; Geller, Davis, & Peterson, 2020). Second, all three experiments employed a mixed-list design. Although perceptual disfluency effects have been observed with both mixed and pure/blocked designs (Geller et al., 2018; Rosner et al., 2015), the mixed-list paradigm is not representative of typical classroom contexts. Third, participants were not informed about the upcoming recognition test. Prior work has shown that low test expectancy can be an important moderator of disfluency effects (Geller & Peterson, 2021). Lastly, while we did not establish a region of practical equivalence (ROPE), the effect sizes observed here appear to be small. Using the default ROPE from the {bayestestR} package (Makowski et al., 2019) (–0.10 to 0.10 in standardized units), many of the critical contrasts for our recognition memory findings fell entirely within or overlapped with this region, suggesting negligible differences. For applied contexts, these effects are likely well below the smallest effect size of interest, but more research is needed.

This is not to say that all research on perceptual disfluency is unwarranted. As emphasized in Geller and Peterson (2021), a promising direction for future work is to examine how perceptual factors influence everyday processing and memory, particularly in situations where encoding is largely incidental (Castel et al., 2015). In addition, educators and researchers might leverage computational modeling approaches to guide the selection of stimuli in more ecologically valid settings, testing whether perceptual disfluency effects can be reliably obtained under conditions that more closely approximate real-world learning environments.

These results also provide some context for the large number of replication failures. Many prior studies on disfluency do not carefully ensure that the manipulation is, in fact, experienced as disfluent. More often than not, such work relies on simple two-level manipulations (fluent vs. disfluent) and employs analytic approaches that may not be well suited to the structure of response time data. As we have attempted to demonstrate here, it is crucial to consider the entire RT distribution. By examining distributional shifts across different levels of disfluency, we obtained a richer understanding of the processing stages affected during encoding. We hope that researchers in learning and memory will increasingly adopt these tools, not only to clarify the mechanisms underlying perceptual disfluency effects, but also to investigate encoding in contexts characterized by substantial cognitive conflict.

## Conclusion

Our paper contributes nuanced insights to the intricate relationship between perceptual disfluency and memory encoding. We have shown that perceptual disfluency can aid in memory retention, but its efficacy is contingent upon the degree of disfluency and other contextual factors such as word frequency. Our findings endorse the stage-specific account, emphasizing the role of cognitive control mechanisms in the observed memory advantages with perceptual disfluency. Furthermore, our methodological contributions, employing an ex-Gaussian model and DDM, not only validate the benefits of examining RT distributions, but also open new avenues for future research in learning and memory studies. We caution, however, that the applicability of these findings in real-world educational settings remains an open question, and the effect sizes observed were relatively small, thus warranting further investigation. Ultimately, this work stands as a call to action for a more comprehensive, nuanced approach to studying perceptual disfluency, incorporating both advanced statistical methods and a more exhaustive range of experimental conditions to better elucidate when and how disfluency can facilitate memory.

## Data Accessibility Statement

Experiment 1A and 2 were preregistered: https://osf.io/q3fjn; https://osf.io/kjq3t. Data, code, and materials for this manuscript can be found at https://osf.io/6sy7k/. The files needed to reproduce this manuscript are located here: https://github.com/jgeller112/Disfluency_Ms.

## Footnotes

To promote transparency and reproducibility, this paper was written in *R* (R Core Team, 2025) using Quarto (Allaire et al., 2024), an open-source publishing system that allows for dynamic and static documents. This system allows figures, tables, and text to be programmatically included directly in the manuscript, ensuring that all results are seamlessly integrated into the document. We used the *rix* (Rodrigues & Baumann, 2025) package which harnesses the power of the nix (Dolstra & contributors, 2023) ecosystem to help with computational reproducibility. This package captures not only the *R* packages used to generate the manuscript but also the system dependencies at run-time. As a result, others can easily reproduce the environment by installing the Nix package manager and using the included *default.nix* file. The *README* file in the GitHub repository contains detailed information on how to set up and reproduce the contents of the current manuscript. We have also included a video tutorial. We hope these supplemental materials will make it easier for researchers to apply this code to their own research. The paper files can be found here: https://github.com/jgeller112/Disfluency_Ms.

This is supported by the {brms} package developer and is discussed in this Github post: https://github.com/paul-buerkner/brms/issues/311. However, there is much debate on the use of BFs.

The parameterization used by {brms} does not match the standard formulation of the ex-Gaussian distribution. In the parameterization used by {brms} *μ* is the mean of the entire distribution, not just the Gaussian part. A custom script was implemented to recover the traditional parameters: https://osf.io/b352t.

## Appendix

The purpose of this appendix is to provide a brief description of how the DDM accounts for the data presented in the main article. Although fitting the diffusion model was included in our preregistration plan, we chose to present these analyses here rather than in the main text to enhance readability.

### DDM Results

We fit a hierarchical Bayesian Wiener diffusion model in {brms} (Vandekerckhove et al., 2011) with accuracy coding, estimating drift rate (*v*), boundary separation (response caution; fixed at 0.5), and non-decision time (*T*_er_).

#### Experiment 1A – DDM Results

**Table A1:** Posterior distribution estimates for DDM (Experiment 1A).

HYPOTHESIS	PARAMETER	MEAN	SE	CrI*	ER	POSTERIOR PROB

High-blur > (Low-blur + Clear)	v	–2.22	0.09	[–2.369, –2.073]	Inf	1.00

low-blur = Clear	v	0.00	0.05	[–0.091, 0.096]	29.65	0.97

High-blur > (Low-blur + Clear)	*T_er_*	0.10	0.00	[0.092, 0.106]	Inf	1.00

Low-blur > Clear	*T_er_*	0.01	0.00	[0.008, 0.018]	Inf	1.00

*Note*. CrI: 90% for one-sided tests and 95% for two-sided tests against 0. Posterior probability indicates the proportion of the posterior distribution that falls on one side of zero (either positive or negative), representing the probability that the effect is greater than or less than zero.

A summary of the diffusion model results can be found in Table A1. There was strong evidence that high-blurred words were associated with a lower drift rate than both clear and low-blurred words, *b* = –2.219, 90% CrI [–2.369, –2.073], ER = Inf. In contrast, the evidence supported the absence of a drift rate difference between low-blurred and clear words, *b* = 0.003, 90% CrI [–0.091, 0.096], ER = 29.651. There was also substantial evidence that non-decision time was greater for high-blurred words compared to both clear and low-blurred words, *b* = 0.099, 90% CrI [0.092, 0.106], ER = Inf. Additionally, the posterior distribution indicated that low-blurred words had a longer non-decision time than clear words, *b* = 0.013, 90% CrI [0.008, 0.018], ER = Inf.

#### Experiment 1B – DDM Results

**Table A2:** Posterior distribution estimates for DDM (Experiment 1B).

HYPOTHESIS	PARAMETER	MEAN	SE	CrI*	ER	POSTERIOR PROB

High-blur < (low-blur + Clear)	v	–0.90	0.06	[–1.001, –0.794]	Inf	1.00

Low-blur = Clear	v	–0.02	0.06	[–0.13, 0.086]	22.89	0.96

High-blur > (Low-blur + Clear)	*T_er_*	0.10	0.01	[0.093, 0.108]	Inf	1.00

Low-blur > Clear	*T_er_*	0.01	0.00	[0.009, 0.02]	Inf	1.00

*Note*. CrI: 90% for one-sided tests and 95% for two-sided tests against 0. Posterior probability indicates the proportion of the posterior distribution that falls on one side of zero (either positive or negative), representing the probability that the effect is greater than or less than zero.

A summary of the DDM results is presented in Table A2. There was strong evidence that high-blurred words were associated with a lower drift rate than both clear and low-blurred words, *b* = –0.896, 90% CrI [–1.001, –0.794], ER = Inf. In contrast, the evidence supported the absence of a drift rate difference between low-blurred and clear words, *b* = –0.023, 90% CrI [–0.13, 0.086], ER = 22.891. There was also substantial evidence that non-decision time was greater for high-blurred words compared to both clear and low-blurred words, *b* = 0.100, 90% CrI [0.093, 0.108], ER = Inf. Additionally, the posterior distribution indicated that low-blurred words had a longer non-decision time than clear words, *b* = 0.014, 90% CrI [0.009, 0.02], ER = Inf.

## References

[1] Allaire, J. J., Teague, C., Scheidegger, C., Xie, Y., Dervieux, C., & Woodhull, G. (2024). Quarto (Version 1.6) [Computer software]. 10.5281/zenodo.5960048

[2] Alter, A. L., Oppenheimer, D. M., Epley, N., & Eyre, R. N. (2007). Overcoming intuition: Metacognitive difficulty activates analytic reasoning. Journal of Experimental Psychology: General, 136(4), 569–576. 10.1037/0096-3445.136.4.569 17999571

[3] Andrews, S., & Heathcote, A. (2001). Distinguishing common and task-specific processes in word identification: A matter of some moment? Journal of Experimental Psychology: Learning, Memory, and Cognition, 27(2), 514–544. 10.1037/0278-7393.27.2.514 11294447

[4] Balota, D. A., & Spieler, D. H. (1999). Word frequency, repetition, and lexicality effects in word recognition tasks: Beyond measures of central tendency. Journal of Experimental Psychology: General, 128(1), 32–55. 10.1037/0096-3445.128.1.32 10100390

[5] Balota, D. A., & Yap, M. J. (2011). Moving beyond the mean in studies of mental chronometry the power of response time distributional analyses. Current Directions in Psychological Science, 20(3), 160–166. http://cdp.sagepub.com/content/20/3/160.short

[6] Balota, D. A., Yap, M. J., Cortese, M. J., & Watson, J. M. (2008). Beyond mean response latency: Response time distributional analyses of semantic priming. Journal of Memory and Language, 59(4), 495–523. 10.1016/j.jml.2007.10.004

[7] Balota, D. A., Yap, M. J., Hutchison, K. A., Cortese, M. J., Kessler, B., Loftis, B., Neely, J. H., Nelson, D. L., Simpson, G. B., & Treiman, R. (2007). The English Lexicon Project. Behavior Research Methods, 39(3), 445–459. 10.3758/bf03193014 17958156

[8] Barnhart, A. S., & Goldinger, S. D. (2010). Interpreting chicken-scratch: Lexical access for handwritten words. Journal of Experimental Psychology: Human Perception and Performance, 36(4), 906–923. 10.1037/a0019258 20695708 PMC4241396

[9] Barr, D. J., Levy, R., Scheepers, C., & Tily, H. J. (2013). Random effects structure for confirmatory hypothesis testing: Keep it maximal. Journal of Memory and Language, 68(3), 255–278. 10.1016/j.jml.2012.11.001 PMC3881361 24403724

[10] Barthelme, S. (2023). Imager: Image processing library based on ‘CImg’. https://CRAN.R-project.org/package=imager

[11] Besken, M., & Mulligan, N. W. (2013). Easily perceived, easily remembered? Perceptual interference produces a double dissociation between metamemory and memory performance. Memory & Cognition, 41(6), 897–903. http://link.springer.com/article/10.3758/s13421-013-0307-8 23460317 10.3758/s13421-013-0307-8

[12] Bjork, E. L., & Bjork, R. A. (2011). Making things hard on yourself, but in a good way: Creating desirable difficulties to enhance learning. In Psychology and the real world: Essays illustrating fundamental contributions to society. (pp. 56–64). Worth Publishers.

[13] Boag, R. J., Strickland, L., Heathcote, A., Neal, A., & Loft, S. (2019). Cognitive control and capacity for prospective memory in complex dynamic environments. Journal of Experimental Psychology: General, 148(12), 2181–2206. 10.1037/xge0000599 31008627

[14] Boag, R. J., Strickland, L., Loft, S., & Heathcote, A. (2019). Strategic attention and decision control support prospective memory in a complex dual-task environment. Cognition, 191, 103974. 10.1016/j.cognition.2019.05.011 31234118

[15] Braver, T. S. (2012). The variable nature of cognitive control: a dual mechanisms framework. Trends in Cognitive Sciences, 16(2), 106–113. 10.1016/j.tics.2011.12.010 22245618 PMC3289517

[16] Bürkner, P.-C. (2017). Brms: An r package for bayesian multilevel models using stan. 80. 10.18637/jss.v080.i01

[17] Carpenter, S. K., Pan, S. C., & Butler, A. C. (2022). The science of effective learning with spacing and retrieval practice. Nature Reviews Psychology, 1(9), 496–511. 10.1038/s44159-022-00089-1

[18] Castel, A. D., Nazarian, M., & Blake, A. B. (2015). Attention and incidental memory in everyday settings (pp. 463–483). Boston Review. 10.7551/mitpress/10033.003.0023

[19] Coltheart, M., Rastle, K., Perry, C., Langdon, R., & Ziegler, J. (2001). DRC: A dual route cascaded model of visual word recognition and reading aloud. Psychological Review, 108(1), 204–256. 10.1037/0033-295X.108.1.204 11212628

[20] Cushing, C., & Bodner, G. E. (2022). Reading aloud improves proofreading (but using sans forgetica font does not). Journal of Applied Research in Memory and Cognition, 11, 427–436. 10.1037/mac0000011

[21] De Jong, R., Liang, C. C., & Lauber, E. (1994). Conditional and unconditional automaticity: a dual-process model of effects of spatial stimulus-response correspondence. Journal of Experimental Psychology. Human Perception and Performance, 20(4), 731–750. 10.1037//0096-1523.20.4.731 8083631

[22] DeCarlo, L. T. (1998). Signal detection theory and generalized linear models. Psychological Methods, 3(2), 186–205. 10.1037/1082-989X.3.2.186

[23] Diana, R. A., & Reder, L. M. (2006). The low-frequency encoding disadvantage: Word frequency affects processing demands. Journal of Experimental Psychology: Learning, Memory, and Cognition, 32(4), 805–815. 10.1037/0278-7393.32.4.805 16822148 PMC2387211

[24] Diemand-Yauman, C., Oppenheimer, D. M., & Vaughan, E. B. (2011). Fortune favors the: Effects of disfluency on educational outcomes. Cognition, 118(1), 111–115. 10.1016/j.cognition.2010.09.012 21040910

[25] Dolstra, E., & contributors, T. N. (2023). Nix (Version 2.15.3) [Computer software]. https://nixos.org/

[26] Earp, J. (2018, October 8). Q&a: Designing a font to help students remember key information. https://www.teachermagazine.com/au_en/articles/qa-designing-a-font-to-help-students-remember-key-information

[27] Eskenazi, M. A., & Nix, B. (2021). Individual differences in the desirable difficulty effect during lexical acquisition. Journal of Experimental Psychology: Learning, Memory, and Cognition, 47(1), 45–52. 10.1037/xlm0000809 31916830

[28] Fernández-López, M., Gómez, P., & Perea, M. (2022). Letter rotations: through the magnifying glass and What evidence found there. Language, Cognition and Neuroscience, 38(2), 127–138. 10.1080/23273798.2022.2093390

[29] Fitousi, D. (2020a). Decomposing the composite face effect: Evidence for non-holistic processing based on the ex-Gaussian distribution. Quarterly Journal of Experimental Psychology, 73(6), 819–840. 10.1177/1747021820904222 31952449

[30] Fitousi, D. (2020b). Linking the ex-gaussian parameters to cognitive stages: Insights from the linear ballistic accumulator (LBA) model. The Quantitative Methods for Psychology, 16(2), 91–106. 10.20982/tqmp.16.2.p091

[31] Gagl, B., Sassenhagen, J., Haan, S., Gregorova, K., Richlan, F., & Fiebach, C. J. (2020). An orthographic prediction error as the basis for efficient visual word recognition. NeuroImage, 214, 116727. 10.1016/j.neuroimage.2020.116727 32173410 PMC7284316

[32] Geller, J., Davis, S. D., & Peterson, D. J. (2020). Sans forgetica is not desirable for learning. Memory, 28(8), 957–967. 10.1080/09658211.2020.1797096 32723219

[33] Geller, J., & Peterson, D. (2021). Is this going to be on the test? Test expectancy moderates the disfluency effect with sans forgetica. Journal of Experimental Psychology: Learning, Memory, and Cognition, 47(12), 1924–1938. 10.1037/xlm0001042 34672664

[34] Geller, J., Still, M. L., Dark, V. J., & Carpenter, S. K. (2018). Would disfluency by any other name still be disfluent? Examining the disfluency effect with cursive handwriting. Memory and Cognition, 46(7), 1109–1126. 10.3758/s13421-018-0824-6 29916114

[35] Glanzer, M., & Adams, J. K. (1985). The mirror effect in recognition memory. Memory & Cognition, 13(1), 8–20. 10.3758/bf03198438 4010518

[36] Gomez, P., & Perea, M. (2014). Decomposing encoding and decisional components in visual-word recognition: A diffusion model analysis. Quarterly Journal of Experimental Psychology, 67(12), 2455–2466. 10.1080/17470218.2014.937447 25192455

[37] Gomez, P., Perea, M., & Ratcliff, R. (2013). A diffusion model account of masked versus unmasked priming: Are they qualitatively different? Journal of Experimental Psychology: Human Perception and Performance, 39(6), 1731–1740. 10.1037/a0032333 23647337 PMC5688948

[38] Grant, R. L., Carpenter, B., Furr, D. C., & Gelman, A. (2017). Introducing the StataStan Interface for Fast, Complex Bayesian Modeling Using Stan. The Stata Journal: Promoting Communications on Statistics and Stata, 17(2), 330–342. 10.1177/1536867x1701700205

[39] Green, D. M., & Swets, J. A. (1966). Signal detection theory and psychophysics. John Wiley.

[40] Halamish, V. (2018). Can very small font size enhance memory? Memory & Cognition, 46(6), 979–993. 10.3758/s13421-018-0816-6 29725876

[41] Heathcote, A., Popiel, S. J., & Mewhort, D. J. (1991). Analysis of response time distributions: An example using the Stroop task. Psychological Bulletin, 109(2), 340–347. 10.1037/0033-2909.109.2.340

[42] Hu, X., Yang, C., & Luo, L. (2022). Retrospective confidence rating about memory performance is affected by both retrieval fluency and non-decision time. Metacognition and Learning, 17(2), 651–681. 10.1007/s11409-022-09303-0

[43] Huff, M. J., Maxwell, N. P., & Mitchell, A. (2022). Distinctive Sans Forgetica font does not benefit memory accuracy in the DRM paradigm. Cognitive Research: Principles and Implications, 7(1). 10.1186/s41235-022-00448-9 PMC9733772 36484976

[44] James, W. (1890). The principles of psychology, vol i. Henry Holt; Co. 10.1037/10538-000

[45] Johnson, R. L., Staub, A., & Fleri, A. M. (2012). Distributional analysis of the transposed-letter neighborhood effect on naming latency. Journal of Experimental Psychology: Learning, Memory, and Cognition, 38(6), 1773–1779. 10.1037/a0028222 22563634

[46] Kane, M. J., & Engle, R. W. (2003). Working-memory capacity and the control of attention: The contributions of goal neglect, response competition, and task set to Stroop interference. Journal of Experimental Psychology: General, 132(1), 47–70. 10.1037/0096-3445.132.1.47 12656297

[47] Kuchinke, L., Vo, M., Hofmann, M., & Jacobs, A. (2007). Pupillary responses during lexical decisions vary with word frequency but not emotional valence. International Journal of Psychophysiology, 65(2), 132–140. 10.1016/j.ijpsycho.2007.04.004 17532075

[48] Lehmann, J., Goussios, C., & Seufert, T. (2015). Working memory capacity and disfluency effect: an aptitude-treatment-interaction study. Metacognition and Learning, 11(1), 89–105. 10.1007/s11409-015-9149-z

[49] Lenth, R. V. (2023). Emmeans: Estimated marginal means, aka least-squares means. https://CRAN.R-project.org/package=emmeans

[50] Makowski, D., Ben-Shachar, M. S., & Lüdecke, D. (2019). bayestestR: Describing effects and their uncertainty, existence and significance within the bayesian framework. 4, 1541. 10.21105/joss.01541 PMC6914840 31920819

[51] Malmberg, K. J., & Nelson, T. O. (2003). The word frequency effect for recognition memory and the elevated-attention hypothesis. Memory & Cognition, 31(1), 35–43. 10.3758/bf03196080 12699141

[52] Mathot, S. (2018). Pupillometry: Psychology, physiology, and function. Journal of Cognition, 1(1). 10.5334/joc.18 PMC6634360 31517190

[53] Matzke, D., & Wagenmakers, E.-J. (2009). Psychological interpretation of the ex-gaussian and shifted wald parameters: A diffusion model analysis. Psychonomic Bulletin & Review, 16(5), 798–817. http://link.springer.com/article/10.3758/PBR.16.5.798 19815782 10.3758/PBR.16.5.798

[54] McClelland, J. L., & Rumelhart, D. E. (1981). An interactive activation model of context effects in letter perception: I. An account of basic findings. Psychological Review, 88(5), 375–407. 10.1037/0033-295X.88.5.375 7058229

[55] McElreath, R. (2020). Statistical rethinking: A bayesian course with examples in r and stan (2nd ed.). CRC Press. 10.1201/9780429029608

[56] Mulligan, N. W. (1996). The effects of perceptual interference at encoding on implicit memory, explicit memory, and memory for source. Journal of Experimental Psychology: Learning, Memory, and Cognition, 22(5), 1067–1087. 10.1037/0278-7393.22.5.1067 8805816

[57] Myers, C. E., Interian, A., & Moustafa, A. A. (2022). A practical introduction to using the drift diffusion model of decision-making in cognitive psychology, neuroscience, and health sciences. Frontiers in Psychology, 13. https://www.frontiersin.org/articles/10.3389/fpsyg.2022.1039172 10.3389/fpsyg.2022.1039172 PMC9784241 36571016

[58] Nairne, J. S. (1988). A framework for interpreting recency effects in immediate serial recall. Memory & Cognition, 16(4), 343–352. 10.3758/BF03197045 3062314

[59] Pazzaglia, A. M., Staub, A., & Rotello, C. M. (2014). Encoding time and the mirror effect in recognition memory: Evidence from eyetracking. Journal of Memory and Language, 75, 77–92. 10.1016/j.jml.2014.05.009

[60] Peirce, J., Gray, J. R., Simpson, S., MacAskill, M., Höchenberger, R., Sogo, H., Kastman, E., & Lindeløv, J. K. (2019). PsychoPy2: Experiments in behavior made easy. Behavior Research Methods, 51(1), 195–203. 10.3758/s13428-018-01193-y 30734206 PMC6420413

[61] Perea, M., Fernández-López, M., & Marcet, A. (2018). Does CaSe-MiXinG disrupt the access to lexico-semantic information? Psychological Research, 84(4), 981–989. 10.1007/s00426-018-1111-7 30370458

[62] Perea, M., Gil-López, C., Beléndez, V., & Carreiras, M. (2016). Do handwritten words magnify lexical effects in visual word recognition? Quarterly Journal of Experimental Psychology, 69(8), 1631–1647. 10.1080/17470218.2015.1091016 26340587

[63] Perea, M., & Lupker, S. J. (2003). Transposed-letter confusability effects in masked form priming. Masked Priming: State of the Art, 97–120. http://defiant.ssc.uwo.ca/faculty/lupkerpdfs/Perea%20&%20Lupker,%202003,%20chapter.pdf

[64] Pieger, E., Mengelkamp, C., & Bannert, M. (2016). Metacognitive judgments and disfluency does disfluency lead to more accurate judgments, better control, and better performance? Learning and Instruction, 44, 31–40. 10.1016/j.learninstruc.2016.01.012

[65] Plourde, C. E., & Besner, D. (1997). On the locus of the word frequency effect in visual word recognition. Canadian Journal of Experimental Psychology/Revue Canadienne de Psychologie Expérimentale, 51(3), 181–194. 10.1037/1196-1961.51.3.181

[66] Ptok, M. J., Hannah, K. E., & Watter, S. (2020). Memory effects of conflict and cognitive control are processing stage-specific: evidence from pupillometry. Psychological Research, 85(3), 1029–1046. 10.1007/s00426-020-01295-3 32036444

[67] Ptok, M. J., Thomson, S. J., Humphreys, K. R., & Watter, S. (2019). Congruency encoding effects on recognition memory: A stage-specific account of desirable difficulty. Frontiers in Psychology, 10. 10.3389/fpsyg.2019.00858 PMC6491626 31068858

[68] R Core Team. (2025). R: A language and environment for statistical computing. R Foundation for Statistical Computing. https://www.R-project.org/

[69] Ratcliff, R. (1978). A theory of memory retrieval. Psychological Review, 85(2), 59–108. 10.1037/0033-295X.85.2.59

[70] Ratcliff, R., Smith, P. L., Brown, S. D., & McKoon, G. (2016). Diffusion Decision Model: Current Issues and History. Trends in Cognitive Sciences, 20(4), 260–281. 10.1016/j.tics.2016.01.007 26952739 PMC4928591

[71] Rhodes, M. G., & Castel, A. D. (2008). Memory predictions are influenced by perceptual information: Evidence for metacognitive illusions. Journal of Experimental Psychology: General, 137(4), 615–625. 10.1037/a0013684 18999356

[72] Rhodes, M. G., & Castel, A. D. (2009). Metacognitive illusions for auditory information: Effects on monitoring and control. Psychonomic Bulletin and Review, 16(3), 550–554. 10.3758/PBR.16.3.550 19451383

[73] Roberts, B. R. T., Hu, Z. S., Curtis, E., Bodner, G. E., McLean, D., & MacLeod, C. M. (2023). Reading text aloud benefits memory but not comprehension. Memory & Cognition. 10.3758/s13421-023-01442-2 37440162

[74] Rodrigues, B., & Baumann, P. (2025). Rix: Reproducible data science environments with ‘nix’. https://docs.ropensci.org/rix/

[75] Roediger, H. L., & Karpicke, J. D. (2006). Test-Enhanced Learning. Psychological Science, 17(3), 249–255. 10.1111/j.1467-9280.2006.01693.x 16507066

[76] Rohrer, D., & Taylor, K. (2007). The shuffling of mathematics problems improves learning. Instructional Science, 35(6), 481–498. 10.1007/s11251-007-9015-8

[77] Rosner, T. M., Davis, H., & Milliken, B. (2015). Perceptual blurring and recognition memory: A desirable difficulty effect revealed. Acta Psychologica, 160, 11–22. 10.1016/j.actpsy.2015.06.006 26134415

[78] Rummer, R., Schweppe, J., & Schwede, A. (2015). Fortune is fickle: Null-effects of disfluency on learning outcomes. Metacognition and Learning, 114. http://link.springer.com/article/10.1007/s11409-015-9151-5

[79] Slamecka, N. J., & Graf, P. (1978). The generation effect: Delineation of a phenomenon. Journal of Experimental Psychology: Human Learning and Memory, 4(6), 592–604. 10.1037/0278-7393.4.6.592

[80] Staub, A. (2010). The effect of lexical predictability on distributions of eye fixation durations. Psychonomic Bulletin & Review, 18(2), 371–376. 10.3758/s13423-010-0046-9 21327339

[81] Sternberg, S. (1969). The discovery of processing stages: Extensions of Donders’ method. Acta Psychologica, 30, 276–315. 10.1016/0001-6918(69)90055-9

[82] Sungkhasettee, V. W., Friedman, M. C., & Castel, A. D. (2011). Memory and metamemory for inverted words: Illusions of competency and desirable difficulties. Psychonomic Bulletin and Review, 18(5), 973–978. 10.3758/s13423-011-0114-9 21626231

[83] Taylor, A., Sanson, M., Burnell, R., Wade, K. A., & Garry, M. (2020a). Disfluent difficulties are not desirable difficulties: The (lack of) effect of sans forgetica on memory. Memory, 28(7), 850–857. 10.1080/09658211.2020.1758726 32364830

[84] Taylor, J. E., Beith, A., & Sereno, S. C. (2020b). LexOPS: An R package and user interface for the controlled generation of word stimuli. Behavior Research Methods, 52(6), 2372–2382. 10.3758/s13428-020-01389-1 32394182 PMC7725696

[85] van der Wel, P., & van Steenbergen, H. (2018). Pupil dilation as an index of effort in cognitive control tasks: A review. Psychonomic Bulletin & Review, 25(6), 2005–2015. 10.3758/s13423-018-1432-y 29435963 PMC6267528

[86] Vandekerckhove, J., Tuerlinckx, F., & Lee, M. D. (2011). Hierarchical diffusion models for two-choice response times. Psychological Methods, 16(1), 44–62. 10.1037/a0021765 21299302

[87] Vehtari, A., Gelman, A., Simpson, D., Carpenter, B., & Bürkner, P.-C. (2021). Rank-normalization, folding, and localization: An improved *R̂* for assessing convergence of MCMC (with discussion). Bayesian Analysis, 16(2), 667–718. 10.1214/20-BA1221

[88] Vergara-Martínez, M., Gutierrez-Sigut, E., Perea, M., Gil-López, C., & Carreiras, M. (2021). The time course of processing handwritten words: An ERP investigation. Neuropsychologia, 159, 107924. 10.1016/j.neuropsychologia.2021.107924 34175372

[89] Weissgerber, S. C., & Reinhard, M. A. (2017). Is disfluency desirable for learning? Learning and Instruction, 49, 199–217. 10.1016/j.learninstruc.2017.02.004

[90] Weltman, D., & Eakin, M. (2014). Incorporating unusual fonts and planned mistakes in study materials to increase business student focus and retention. INFORMS Transactions on Education, 15(1), 156–165. 10.1287/ited.2014.0130

[91] Wenzel, K., & Reinhard, M.-A. (2019). Relatively unintelligent individuals do not benefit from intentionally hindered learning: The role of desirable difficulties. Intelligence, 77, 101405. 10.1016/j.intell.2019.101405

[92] Westerman, D. L., & Greene, R. L. (1997). The effects of visual masking on recognition: Similarities to the generation effect. Journal of Memory and Language, 37(4), 584–596. 10.1006/jmla.1997.2531

[93] Wetzler, E. L., Pyke, A. A., & Werner, A. (2021). Sans Forgetica is Not the “Font” of Knowledge: Disfluent Fonts are Not Always Desirable Difficulties. SAGE Open, 11(4), 21582440211056624. 10.1177/21582440211056624

[94] Wilcox, R. R. (1998). How many discoveries have been lost by ignoring modern statistical methods? American Psychologist, 53(3), 300–314. 10.1037/0003-066x.53.3.300

[95] Wit, B. de, & Kinoshita, S. (2015). The masked semantic priming effect is task dependent: Reconsidering the automatic spreading activation process. Journal of Experimental Psychology: Learning, Memory, and Cognition, 41(4), 1062–1075. 10.1037/xlm0000074 25485751

[96] Yap, M. J., & Balota, D. A. (2007). Additive and interactive effects on response time distributions in visual word recognition. Journal of Experimental Psychology: Learning, Memory, and Cognition, 33(2), 274–296. 10.1037/0278-7393.33.2.274 17352611

[97] Yue, C. L., Castel, A. D., & Bjork, R. A. (2013). When disfluency isand is nota desirable difficulty: The influence of typeface clarity on metacognitive judgments and memory. Memory & Cognition, 41(2), 229–241. http://link.springer.com/article/10.3758/s13421-012-0255-8 22976883 10.3758/s13421-012-0255-8

[98] Zloteanu, M., & Vuorre, M. (2024). A Tutorial for Deception Detection Analysis or: How I Learned to Stop Aggregating Veracity Judgments and Embraced Signal Detection Theory Mixed Models. Journal of Nonverbal Behavior, 48(1), 161–185. 10.1007/s10919-024-00456-x

